# The availability, price and affordability of essential antibacterials in Hubei province, China

**DOI:** 10.1186/s12913-018-3835-x

**Published:** 2018-12-29

**Authors:** Guangjie Wu, Shiwei Gong, Hongbing Cai, Yufeng Ding

**Affiliations:** 1 Department of Pharmacy, Tongji Hospital, Tongji Medical College, Huazhong University of Science and Technology, Wuhan, 430030 Hubei People’s Republic of China; 2 Tongji Hospital, No.1095, Jiefang Avenue, Wuhan, Hubei province China; 3 Department of Pharmacy Business and Administration; School of Pharmacy; Tongji Medical College, Huazhong University of Science and Technology, Wuhan, 430030 Hubei People’s Republic of China; 4 Tongji Medical College, No.13, Jiefang Avenue, Wuhan, Hubei province China

**Keywords:** Essential antibacterials, Availability, Price, Affordability, China

## Abstract

**Background:**

China ranks first amongst the countries for the abuse of antibacterials. Essential antibacterials could help solve the problem. The aim of the work is to evaluate the availability, price and affordability of essential antibacterials in Hubei province, China.

**Method:**

The standardized methodology developed by the World Health Organization and Health Action International was used to collect data on the availability and prices of 16 antibacterials in 5 cities of Hubei province, China.

**Results:**

First, in total, the median availability of originator brands and lowest-priced generics for the essential antibacterials was low, 3.0% (0.0, 18.2%) and 33.3% (0.0, 87.9%) for each, respectively. Second, the median price ratio of originator brands for the antibacterials was 20.30 (4.71, 35.80), while for generics, it was 0.49 (0.07, 1.18). Third, the affordability of originator brands for the antibacterials was 28.14 (21.70, 41.90) times the daily wages of an unskilled government worker, while for generics, the affordability was 0.35 (0.04, 6.11). Finally, we found that in Hubei province, lowest-priced generics for essential antibacterials with (fairly) high availability and relatively low price included Amoxicillin/Clavulanic Acid, Ceftazidime, Metronidazole, Gentamicin Sulfate and Ceftriaxone.

**Conclusion:**

The prices of lowest-priced generics for essential antibacterials in Hubei province were reasonable, and in tertiary hospitals the availability was the highest, while in secondary and primary hospitals, it was relatively lower. Originator brands were not only extremely expensive but also difficult to obtain. Measures should be taken to improve the availability of essential antibacterials and the affordability of originator brands.

**Electronic supplementary material:**

The online version of this article (10.1186/s12913-018-3835-x) contains supplementary material, which is available to authorized users.

## Background

Antibacterials are medicines that are used to prevent and treat bacterial infections [1, 2]. Ever since penicillin was introduced into medical therapy, hundreds of antibacterials have been isolated or synthesized to save lives from infectious diseases [3]. As more and more antibacterials are provided, overuse and misuse of them have been a serious problem. The overuse and misuse of antibacterials stimulated more rapid growth of antibacterial-resistance bacteria, reducing their therapeutic potential against human and animal pathogens [4]. World Health Organization(WHO) characterises antimicrobial resistance as a global public health crisis which must be managed with the utmost urgency [5]. According to the WHO, overuse of antibacterials will cause 10 million deaths and a loss of 100,000 billion US dollars by 2050 [6].

Overusing and misusing antibacterials is a key driver of bacteria resistance. When a new antibacterial is introduced into medical usage, drug resistance to it follows naturally, but it sometimes comes swiftly [7]. Overusing antibacterials concerns more about consumption. China had the largest consumer group of antibacterials use besides Brazil, Russia, India and South Africa [8]. In 2010, China was the second largest consumption of antibacterials with 10.0 × 10 [9] units, following India [8]. While in 2013, 92,700 t of antibacterials were consumed in China, and 48% of which were used in humans [3]. From 2000 to 2010, consumption of antibacterials in hospitals among developing countries had a great increase, in which 57% was attributed to China [8]. As estimated, each Chinese person receives treatment by antibacterials in an average amount of 138 g a year, which is 10 times that consumed in the United States [9]. Further, the rate of antibacterial prescriptions for inpatients is 80%, compare with just 10% in hospitals in high-income countries, while the WHO recommends a maximum of 30% [10, 11]. Problem of antibacterials misuse in China comes as using too many high-level antibacterials instead of essential antibacterials. In Hubei province, essential antibacterials were not fully used, while Cefotaxime, Cefoperazone, Cefamandole, Levofloxacin, Amikacin are preferred (Additional file 1) [12–14]. Similarly, in the newest report, quinolone, 3rd generation cephalosporings, cephalosporings with enzyme inhibitor, 2nd generation cephalosporings, and penicillins with enzyme inhibitor were the 5 kinds of antibacterials that were used most frequently in public hospitals [15].

Owing to the overuse and misuse of antibacterials, China has a rapid growth rate of antibacterial resistance. A high prevalence of antibacterial resistance occurs among healthcare-associated pathogens such as *mycobacterium tuberculosis, Gram-negative organisms and Gram-positive organisms* [16]*.* Most antibacterials except amoxicillin/clavulanic acid, levofloxacin and chloramphenicol, have high resistance [17]. These facts caused worldwide concern. Scientists said that in the foreseeable future, carbapenem resistance in China would be serious [18]. In China, many researchers have focused on the problem of antibacterial overuse and misuse (Additional file 2). Studies showed that even the well-educated groups in China have problems with antibacterial use, we can infer that the situation across China is no more optimistic. The reason for this could be concluded as patients’ expectations, financial incentives from pharmaceutical suppliers, hospital income from drug sale profits and fragmentary, incomplete national guidance on the use of antibacterials that led to antibacterial overuse [19].

Misuse or overuse of antibacterials could lead to severe resistance and loss of money, and choosing effective essential drugs could help reduce overuse of antibacterials and slow down antibacterial resistance [20]. For this purpose, the policy of having a national essential medicine list was rolled out and could help restrain the problem from the source. The policy requires patients to get rational and efficient treatment [21]. Availability and affordability are the two main criteria to assess whether patients can receive timely, adequate and efficient treatment [22]. Until now, several surveys have been conducted to study the availability and affordability of (essential) medicines in China to evaluate the progress of the new policy [21, 23–25]. However, to our knowledge, so far, there has not been a survey to study the availability and affordability of antibacterials alone in Hubei province, so this study aims to determine the high-availability, low-cost antibacterials for three levels of hospitals in Hubei province, based on the most recently published 19th WHO Model List of Essential Medicines to make a suggestion regarding the fight against antibacterial resistance.

## Objective

This study aimed to evaluate the effects of essential medicine policy on the availability of antibacterials in Hubei province and to assess the prices of originator brands (OBs) and lowest-priced generics (LPGs) for essential antibacterials. This study was also conducted to find essential antibacterials with high availability and low price for three levels of hospitals in Hubei province.

## Method

This study was conducted in 33 hospitals from 5 cities, including Wuhan, Yichang, Xiangyang, Jingzhou and Huanggang (Table 1), in Hubei province, central China, from August to October in 2016, using a questionnaire mainly containing the surveyed information of drugs’ common name, dosage, brand name, supplied dosage, price etc. which is developed by the WHO/HAI. Survey data were entered into the pre-programmed Excel Workbook (WHO/HAI 2008) by two people using a double entry technique. Sixteen essential antibacterials were included based on the 19th WHO Model List of Essential Medicines and Essential Medicines List for Hubei province in 2014 (Additional file 3). For each medicine, data on the availability and price of OBs and LPGs were collected. Prices were recorded on the day of the survey. The unit prices were calculated and entered into the workbook.

**Table 1:** Samples of facilities

Districts	Hospitals			
	Primary	Secondary	Tertiary	Total
Wu Han	2	2	2	6
Yi Chang	2	2	2	6
Xiang Yang	2	3	2	7
Jing Zhou	3	2	2	7
Huang Gang	2	4	1	7
Total	11	13	9	33

The availability of each medicine was reported as the percentage of outlets in which the surveyed medicine could be offered on the day of data collection. The median price ratio (MPR), or the ratio of one medicine’s median unit price to the international reference prices (IRPs) according to the Management Sciences for Health (MSH) Price Indicator Guide for 2015, was used for price evaluation. To assess affordability, the ratio of the medicine prices in a standard treatment to the average daily wage of the lowest-paid unskilled government workers could be used. Due to the lack of official data on daily wages of the lowest-paid workers, we used the mean minimum wage in Hubei province instead of it, which was RMB 36.67 (USD 5.50) at the survey time.

## Availability, price and affordability: Criteria in the study

### Availability

- Absent, 0% of hospitals: the medicines were not found in any surveyed hospitals.
- Low, < 50% of hospitals: the medicines were hard to be found.
- Fairly high, 50–80% of hospitals: the medicines were available in many hospitals.
- High, > 80% of hospitals: the medicines have good availability.

### Price

The MPR for each medicine was calculated using the Workbook developed by WHO/HAI, only if the medicine was available in at least one facility. The MPR is the local median unit price of a medicine in comparison with the median unit price found in the Management Sciences for Health (MSH) Price Indicator Guide for 2015 (MSH 2015). The ideal value for MPR was used to represent acceptable local price ratios:

- Acceptable line: MPR < 1;
- Warning line for generics: MPR > 2

According to the WHO/HAI methodology, if an MPR is twice the international reference price for a generic equivalent product (considered a warning line) [26], then this should be a cause for concern.

### Affordability

Affordability was estimated by comparing the total cost of a medicine for a standard course of treatment to the daily wages of the lowest paid unskilled government worker, which was 36.67 CNY per day at the time of the survey.

- Good: Affordability < 1

## Results

### Availability

Generally, both OBs and LPGs of the antibacterials had low availability in the surveyed hospitals, with median availability 3.0% (0.0, 18.2%) for OBs and 33.3% (0.0, 87.9%) for LPGs (Table 2). Among the OBs, Ceftriaxone, Ceftazidime, Ciprofloxacin, Cefaclor and Linezolid could be offered at the study time, though the availability was low, while Amoxicillin/Clavulanic Acid, Ampicillin, Cephalexin and Roxithromycin were absent. Differently, for LPGs, Amoxicillin/Clavulanic Acid and Gentamicin Sulfate had high availability (84.8 and 87.9%), while Linezolid was absent because there were no generic equivalents in China (Table 2 and Table 3).

**Table 2:** Detailed Availability of the surveyed antibacterial in different levels of hospitals

Number	Medicine	LEML	Total Availability		Availability in Primary Hospitals		Availability in Secondary Hospitals		Availability in Tertiary Hospitals	
			OBs	LPGs	OBs	LPGs	OBs	LPGs	OBs	LPGs
1	Amoxicillin/Clavulanic Acid	Y	0.0%	84.8%	0.0%	100.0%	0.0%	76.9%	0.0%	77.8%
2	Ampicillin	Y	0.0%	24.2%	0.0%	45.5%	0.0%	15.4%	0.0%	11.1%
3	Ceftriaxone	Y	12.1%	75.8%	0.0%	90.9%	7.7%	53.8%	33.3%	88.9%
4	Ceftazidime	Y	3.0%	69.7%	0.0%	36.4%	0.0%	84.6%	11.1%	88.9%
5	Chloramphenicol	N	–	3.0%	–	0.0%	–	0.0%	–	11.1%
6	Ciprofloxacin	Y	3.0%	18.2%	0.0%	18.2%	0.0%	15.4%	11.1%	22.2%
7	Nitrofurantoin	N	–	30.3%	–	27.3%	–	30.8%	–	33.3%
8	Cephalexin	Y	0.0%	33.3%	0.0%	18.2%	0.0%	30.8%	0.0%	55.6%
9	Gentamicin Sulfate	N	–	87.9%	–	90.9%	–	76.9%	–	100.0%
10	Norfloxacin	N	–	69.7%	–	36.4%	–	84.6%	–	88.9%
11	Metronidazole	Y	–	78.8%	–	54.5%	–	84.6%	–	100.0%
12	Piperacillin+Tazobactam	Y	–	21.2%	–	18.2%	–	7.7%	–	44.4%
13	Cefaclor	Y	18.2%	33.3%	18.2%	45.5%	0.0%	38.5%	44.4%	11.1%
14	Roxithromycin	Y	0.0%	60.6%	0.0%	63.6%	0.0%	53.8%	0.0%	66.7%
15	Furazolidone	N	–	27.3%	–	18.2%	–	23.1%	–	44.4%
16	Linezolid	N	3.0%	–	0.0%	–	0.0%	–	11.1%	–
Median			3.0%	33.3%	–	36.4%	–	34.7%	11.1%	50.0%

*LEML* Local essential medicine list, *OBs* Originator brands, *LPGs* Lowest-priced generics

**Table 3:** Availability of essential antibacterials in the surveyed hospitals

Availability	Range	Total		Primary Hospitals		Secondary Hospitals		Tertiary Hospitals	
		OBs	LPGs	OBs	LPGs	OBs	LPGs	OBs	LPGs
Absent	0%	Amoxicillin/	Linezolid	The rest	Linezolid	The rest	Chloramphenicol	The rest	Linezolid
		Clavulanic Acid			Chloramphenicol		Linezolid		
		Ampicillin							
		Cephalexin							
		Roxithromycin							
Low	< 50%	Ceftriaxone	Ampicillin	Cefaclor	Ampicillin	Ceftriaxone	Ampicillin	Ceftriaxone	Ampicillin
		Ceftazidime	Chloramphenicol		Ceftazidime		Ciprofloxacin	Ceftazidime	Chloramphenicol
		Ciprofloxacin	Ciprofloxacin		Ciprofloxacin		Nitrofurantoin	Ciprofloxacin	Ciprofloxacin
		Cefaclor	Nitrofurantoin		Nitrofurantoin		Cephalexin	Cefaclor	Nitrofurantoin
		Linezolid	Cephalexin		Cephalexin		Piperacillin+Tazobactam	Linezolid	Piperacillin+Tazobactam
			Piperacillin+Tazobactam		Norfloxacin		Cefaclor		Cefaclor
			Cefaclor		Piperacillin+Tazobactam		Furazolidone		Furazolidone
			Furazolidone		Cefaclor				
					Furazolidone				
Fairly High	50–80%	None	Ceftriaxone	None	Metronidazole	None	Amoxicillin/	None	Amoxicillin/
			Ceftazidime		Roxithromycin		Clavulanic Acid		Clavulanic Acid
			Norfloxacin				Ceftriaxone		Cephalexin
			Metronidazole				Gentamicin Sulfate		Roxithromycin
			Roxithromycin				Roxithromycin		
High	> 80%	None	Amoxicillin/	None	Amoxicillin/	None	Ceftazidime	None	Ceftriaxone
			Clavulanic Acid		Clavulanic Acid		Norfloxacin		Ceftazidime
			Gentamicin Sulfate		Ceftriaxone		Metronidazole		Gentamicin Sulfate
					Gentamicin Sulfate				Norfloxacin
									Metronidazole

*OBs* Originator Brands, *LPGs* Lowest-priced generics

Table 2 shows that the median availabilities of LPGs in primary and secondary hospitals were nearly the same, 36.4% (0.0, 100%) for primary hospitals and 34.7% (0.0, 84.6%) for secondary hospitals. The LPGs with high availability in primary hospitals included Amoxicillin/Clavulanic Acid (100%), Ceftriaxone (90.9%), and Gentamicin Sulfate (90.9%), while Chloramphenicols were completely unavailable. In the secondary hospitals, Ceftazidime, Norfloxacin and Metronidazole had high availability (all 84.6%), while drugs with high availability in the primary hospitals were all in the fairly high-level category. Likewise, LPG of Chloramphenicol was absent in the secondary hospitals. For tertiary hospitals, several OBs of antibacterials could be offered, including Ceftriaxone, Ceftazidime, Ciprofloxacin, and Cefaclor as well as Linezolid, and the median availability was low (11.1%). For LPGs, Ceftriaxone (88.9%), Ceftazidime (88.9%), Gentamicin Sulfate (100.0%), Norfloxacin (88.9%), and Metronidazole (100.0%) had high availability. In addition, the only antibacterial without an LPG was Linezolid, while Chloramphenicol had 11.1% availability.

### Medicine prices

As shown in Table 4, in the surveyed hospitals, the median MPRs for 3 OBs and 11 LPGs were 20.30 (4.71. 35.8) and 0.49 (0.07, 1.18) times their IRPs, respectively. For 3 medicines available as both OBs and LPGs, including Ceftriaxone, Ceftazidime and Ciprofloxacin, OBs cost nearly 30 times more than LPGs. The OB of Ciprofloxacin was extremely expensive in the surveyed hospitals, such that the price was 35.8 times its IRP. All LPGs, except Amoxicillin/Clavulanic Acid and Ciprofloxacin, had lower prices than the IRP. In fact, the MPRs of Amoxicillin/Clavulanic Acid and Ciprofloxacin were both 1.18, below the warning line (MPR = 2.0). The lowest MPR of OBs belonged to Nitrofurantoin (0.07).

**Table 4:** MPR of 11 kinds of antibacterials in the surveyed hospitals

No	Medicine	MPR	
		OBs	LPGs
1	Amoxicillin/Clavulanic Acid	–	1.18
2	Ampicillin	–	0.16
3	Ceftriaxone	20.30	0.43
4	Ceftazidime	4.71	0.69
5	Chloramphenicol	–	0.40
6	Ciprofloxacin	35.8	1.18
7	Nitrofurantoin	–	0.07
8	Cephalexin	–	0.83
9	Gentamicin Sulfate	–	0.49
10	Metronidazole	–	0.67
11	Piperacillin+Tazobactam	–	0.17
Median		20.30	0.49

*MPR* Median price ratio, *OBs* Originator brands, *LPGs* Lowest-priced generics

### Affordability

Table 5 shows the affordability of treatments with 3 OBs and 11 LPGs. All OBs cost more than 1 day’s wages. The median affordability of OBs was 28.14 (21.70, 41.90). In addition, the generic equivalents of the OBs all cost less, with 0.46 for Ceftriaxone (vs. 21.70), 6.11 for Ceftazidime (vs. 41.90), and 0.93 for Ciprofloxacin (vs. 28.14). Generally, choosing LPGs for treatment cost less. The median affordability of LPGs was 0.35 (0.04, 6.11). In fact, only Ceftazidime and Piperacillin/Tazobactam cost more than 1 day’s wages, with affordability of 6.11 and 2.58, respectively, for each.

**Table 5:** Affordability of 11 kinds of antibacterials in the surveyed hospitals

Number	Description	Strength	DDD	Duration Days	Affordability	
					OBs	LPGs
1	Amoxicillin/Clavulanic Acid	500 mg/125 mg	1 g	7	–	0.35
2	Ampicillin	500 mg	2 g	7	–	0.31
3	Ceftriaxone	1 g	2 g	7	21.70	0.46
4	Ceftazidime	1 g	4 g	7	41.90	6.11
5	Chloramphenicol	250 mg	3 g	7	–	0.05
6	Ciprofloxacin	2 mg/ml	0.5 g	7	28.14	0.93
7	Nitrofurantoin	100 mg	0.2 g	7	–	0.04
8	Cephalexin	250 mg	2 g	7	–	0.40
9	Gentamicin Sulfate	40 mg/ml	0.24 g	7	–	0.23
10	Metronidazole	200~250 mg	1.5 g	7	–	0.04
11	Piperacillin+Tazobactam	4 g + 500 mg	14 g	7	–	2.58
Median					28.14	0.35

*DDD* Defined daily dose, *OBs* Originator brands, *LPGs* Lowest-priced generics

### Comprehensive analysis of medicine availability and price

**Fig. 1:**
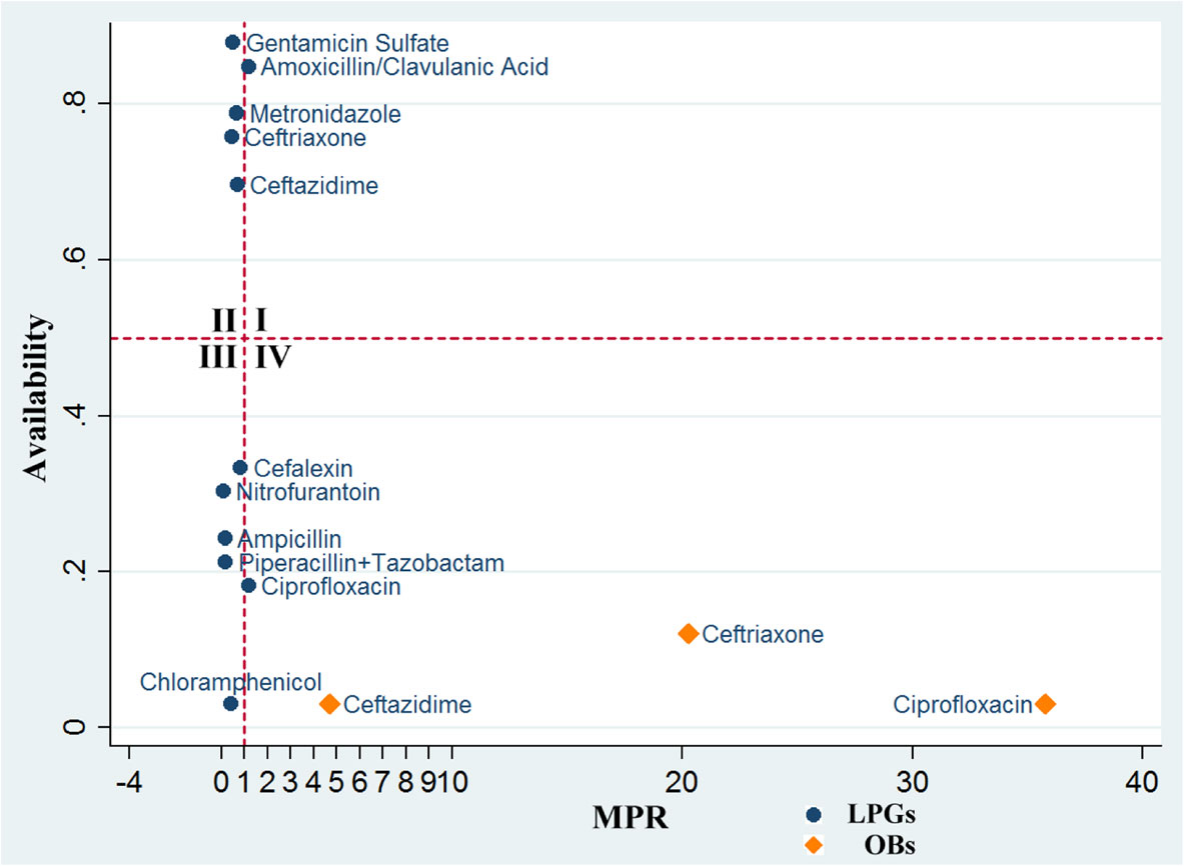
Comprehensive analysis of medicine availability and price. The surveyed antibacterials were put into different quadrants based on their availability and MPRs. Quadrant I: Drugs with high availability and high MPR (price). Quadrant II: Drugs with high availability and low MPR (price). Quadrant III: Drugs with low availability and low MPR (price). Quadrant IV: Drugs with low availability and high MPR (price). Dots stand for lowest-priced generics (LPGs), and squares stand for originator brands

Fig. 1 depicts the availability and prices of both OBs and LPGs for the surveyed antibacterials. For medicines located in the 2nd quadrant, patients had good access and relatively low prices; for example, the LPG for Gentamicin Sulfate had 87.9% availability and an MPR of 0.49. Generics located in the 1st quadrant were medicines with high availability, but with prices higher than the IRPs, including Amoxicillin/Clavulanic Acid. For generics in the 3rd quadrant, the prices were low, but patients had difficulty getting them in the hospitals; for example, though the MPR of Chloramphenicol was 0.40, it only had 3.0% availability. For generics in the 4th quadrant, patients not only had to pay higher prices than the reference prices but were also not able to easily obtain the medicines; for example, the LPG for Ciprofloxacin had 18.2% availability and an MPR of 1.18. In contrast, all LPGs of the surveyed essential antibacterials in Hubei province had MPR values below the warning line (MPR = 2.0). For OBs, the three antibacterials were all located in the 4th quadrant, including Ceftazidime, Ceftriaxone and Ciprofloxacin.

Overall, LPGs of essential antibacterials had reasonable prices, and the availability ranged widely, while OBs were not only difficult to obtain but also expensive.

## Discussion

There are four main findings in this essay. First, in total, the median availabilities of OBs and LPGs for the essential antibacterials were low. The low availability of essential antibacterials could be explained by the following reasons. First, the government’s compensation mechanism was not clear, so hospitals had no willness to use essential medicines, which brought less profit. Second, the public had the opinion that essential medicines were cheap and old drugs, which were less powerful than expensive and new drugs, so hospitals and doctors were pressured against using essential medicines. Third, the research and development ability of pharmaceutical companies grew rapidly, more and more new drugs were advertised, and essential drugs lacked propaganda; as a result, doctors tended to use new drugs with advertised better effects and less adverse actions. Finally, a handful of doctors might be impelled by economic benefits not to prescribe essential medicines for patients, so essential medicines were likely to be eliminated.

Second, the median MPR of OBs for the antibacterials was 20.30 (4.71, 35.80), while for LPGs, it was 0.49 (0.07, 1.18), which was at an acceptable level. In fact, all LPGs for the antibacterials could be acquired with acceptable prices. It was clear that OBs for the surveyed antibacterials were extremely expensive, while prices of generic equivalents were quite acceptable. In fact, in China, the cost of OBs should not exceed LPGs by 35% or more [21], but in our survey, the OB cost at least 6.8 times more than the LPG for Ceftazidime. The high prices of OBs might be due to the bad profit mechanisms when OBs were imported into China. Prices could be elevated several times when crossing levels of related organizations. Luckily, prices for all generics in Hubei province were below the warning line (MPR = 2.0), and only Amoxicillin/Clavulanic Acid and Ciprofloxacin had MPRs > 1. Reported by *Hao Yang* et al. in 2009 [23], MPRs of Amoxicillin and Ciprofloxacin were under 1.5, which was similar to our research. In the survey previously conducted in Shaanxi province [24], which also focused on essential medicines, the authors found that the procured prices of 28 generics were 0.75 times their reference prices. This finding reveals that the prices of generics for essential medicines were low and reasonable. It was not surprising that LPGs for essential antibacterials were cheap because the cost of generics in research and development was omitted. Thus, government should intensify price negotiations with pharmaceutical companies to decrease OB prices and take measures to supervise the abnormal price increases.

Third, the affordability of OBs for the antibacterials was 28.14 (21.70, 41.90) times the daily wage of an unskilled government worker, while for LPGs, the affordability was 0.35 (0.04, 6.11) times the daily wage. Due to the high price of OBs, the affordability of OBs was poor. Lowering the prices of OBs is extremely urgent. On the other hand, in general, the affordability of LPGs was quite reasonable. All but Ceftazidime and Piperacillin/Tazobactam had affordability below 1. According to Table 2, prices of LPGs for these two antibacterials were less than their international reference prices, so the prices were acceptable. Hence, for Ceftazidime and Piperacillin/Tazobactam, government should increase the insurance and payment reimbursement ratio to improve the affordability of these two medicines.

Finally, we found that in Hubei province, LPGs for the essential antibacterials with (fairly) high availability and relatively low price included Amoxicillin/Clavulanic Acid, Ceftazidime, Metronidazole, Gentamicin Sulfate and Ceftriaxone. These essential medicines could cover most pathogens in common infections. We suggest that doctors and decision-makers should consider using these essential antibacterials more.

## Conclusion

Chinese NEML policy requires every public hospital to stock essential medicines, including antibacterials, but the availability in Hubei province was still low. The high prices and poor affordability of originator brands might influence their use. Optimistically, the lowest-priced generics might make up for this situation, but more things still need to be done. Choosing essential antibacterials is a way to promote rational drug use because it excludes profit factors, and using the appropriate essential antibacterials could help decrease high-level antibacterial overuse with the purpose of reducing antibacterial resistance.

## Additional files

Additional file 1: The list of common antibacterials. Representative examples of common antibacterials classified by their therapeutic class and the most frequently used kinds in Hubei province. (DOCX 16 kb)

Additional file 2: Articles about misuse of antibacterials in China. A brief review of articles about misuse of antibacterials in China, according to the published years [26–31]. (DOCX 16 kb)

Additional file 3: The list of surveyed antibacterials. List of the surveyed medicines including their therapeutic class, name, type, dosage form, and belonged catalogues. (DOCX 16 kb)

Additional file 4: International Ethical Guidelines for Biomedical Research Involving Human Subjects. A guideline of how ethics committee censors research on human subjects. (PDF 166 kb)

## Abbreviations

HAI: Health Action International
IRP: International Reference Price
LPGs: Lowest-Priced Generics
MPR: Median Price Ratio
NEML: National Essential Medicine List
OBs: Originator Brands
R&D: Research and Development
WHO: World Health Organization
