# Supplementary material for: The availability, price and affordability of essential antibacterials in Hubei province, China
Source: BMC Health Serv Res. 2018 Dec 29;18:1013. doi: 10.1186/s12913-018-3835-x (PMC6310993; doi:10.1186/s12913-018-3835-x)
Supplement: Supplementary file 1 — The list of common antibacterials. Representative examples of common antibacterials classified by their therapeutic class and the most frequently used kinds in Hubei province. (DOCX 16 kb) [file 12913_2018_3835_MOESM1_ESM.docx]

| **Therapeutic Class** | **Representative**  **Drugs** | **Essential** | **Preferred** |
| --- | --- | --- | --- |
| **1st generation cephalosporins** | Cefazolin Cefradine Cefathiamidine  Ceftezole | Cephalexin | Cefuroxime  Cefoperazone  Ceftriaxone  Cefotaxime  Levofloxacin  Ofloxacin  Cefamandole  Amikacin |
| **2nd generation cephalosporins** | Cefuroxime  Cefamandole | Ceftazidime  Cefaclor |  |
| **3rd generation cephalosporins** | Cefotaxime  Ceftizoxime  Cefoperazone  Cefpiramide  Cefodizime  Cefminox | Ceftriaxone |  |
| **4th generation cephalosporins** | Cefepime  Cefpirome |  |  |
| **Penicillins** | Benzyl penicillin  Azlocillin | Ampicillin Amoxicillin/Clavulanic Acid Piperacillin/Tazobactam |  |
| **Quinolone** | Levofloxacin Pefloxacin  Ofloxacin | Ciprofloxacin  Norfloxacin |  |
| **Aminoglycosides** | Amikacin  Netilmicin  Tobramycin | Gentamicin Sulfate |  |
| **Macrolides** | Erythromycin  Clarithromycin  Azithromycin | Roxithromycin |  |
| **Nitroimidazole** | Ornidazole | Metronidazole |  |
| **Amphenicols** |  | Chloramphenicol |  |
| **Nitrofuran** |  | Nitrofurantoin  Furazolidone |  |
| **Oxazolidinones** |  | Linezolid |  |
