# Supplementary material for: The availability, price and affordability of essential antibacterials in Hubei province, China
Source: BMC Health Serv Res. 2018 Dec 29;18:1013. doi: 10.1186/s12913-018-3835-x (PMC6310993; doi:10.1186/s12913-018-3835-x)
Supplement: Supplementary file 2 — Articles about misuse of antibacterials in China. A brief review of articles about misuse of antibacterials in China, according to the published years [26–31]. (DOCX 16 kb) [file 12913_2018_3835_MOESM2_ESM.docx]

| **Year** | **Author** | **Region** | **Objective** | **Important Result** |
| --- | --- | --- | --- | --- |
| 2014 | Lv B et.al^26^ | Xi’an Jiaotong University, China | To evaluate knowledge of self-medication with antibacterials among university students | 40.2% of students had self-medication with antibacterials, and 59.2% had no prescriptions. |
| 2015 | Zhu X et.al^27^ | Jiangsu University, China | To evaluate self-medication practices with antibacterials among university students | 47.9% of students had a self-medication with antibacterials history, 73.5% self-medicated with at least two different antibacterials. |
| 2016 | Wang XM et. al^28^ | 6 universities in 6 provinces, China (Zhejiang, Wuhan, Nankai, Jilin, Guizhou and Lanzhou) | To explore knowledge and behaviors of university students on antibacterials use | Only 2% of the students answered all 13 questions about antibacterials correctly. 66% of the students tried to buy antibacterials without a prescription and 96% succeeded. |
| 2017 | Wang XM et. al^29^ | 6 universities in 6 provinces | To study antibacterials misuse for self-limiting illnesses | 65.7% of students who went to a doctor with self-limiting illnesses were prescribed antibacterials. |
| 2017 | Chang J et.al^30^ | Nanjing (eastern), Changsha (central), Xi’an(western), China. | To quantify retail pharmacies sales of antibiotics without a medical prescription | Of 256 pharmacies, antibacterials were obtained without a prescription from 55.9% (95%CI 49.5%-62.0%). |
| 2017 | Hu Y et.al^31^ | 6 universities, China | To study antibacterials use in Chinese medical students | 54% of the surveyed medical students had self-medication, 64% stocked antibacterials and 57.7% received antibacterials with self-limiting illnesses. |
