# Supplementary material for: The availability, price and affordability of essential antibacterials in Hubei province, China
Source: BMC Health Serv Res. 2018 Dec 29;18:1013. doi: 10.1186/s12913-018-3835-x (PMC6310993; doi:10.1186/s12913-018-3835-x)
Supplement: Supplementary file 3 — The list of surveyed antibacterials. List of the surveyed medicines including their therapeutic class, name, type, dosage form, and belonged catalogues. (DOCX 16 kb) [file 12913_2018_3835_MOESM3_ESM.docx]

| **Therapeutic Class** | **Generic drug name** | **Type** | **Form** | **WHO**  **EML** | **EML of Hubei**  **province** |
| --- | --- | --- | --- | --- | --- |
| β-Lactam | Amoxicillin/  Clavulanic Acid | OB/LPG | Oral | Y | Y |
|  | Ampicillin | OB/LPG | Injection | Y | Y |
|  | Ceftriaxone | OB/LPG | Injection | Y | Y |
|  | Ceftazidime | OB/LPG | Injection | Y | Y |
|  | Cephalexin | OB/LPG | Oral | Y | Y |
|  | Piperacillin/  Tazobactam | LPG | Injection | Y | Y |
|  | Cefaclor | OB/LPG | Oral | Y | Y |
| Amphenicols | Chloramphenicol | LPG | Oral | Y | N |
| Quinolones | Ciprofloxacin | OB/LPG | Injection | Y | Y |
|  | Norfloxacin | LPG | Oral | Y | N |
| Nitrofuran | Nitrofurantoin | LPG | Oral | Y | N |
|  | Furazolidone | LPG | Oral | Y | N |
| Aminoglycosides | Gentamicin Sulfate | LPG | Injection | Y | N |
| Nitroimidazole | Metronidazole | LPG | Oral | Y | Y |
| Macrolides | Roxithromycin | OB/LPG | Oral | Y | Y |
| Oxazolidinones | Linezolid | OB | Oral | Y | N |

**Notes: EML: Essential medicine list OBs: Originator brands LPGs: Lowest-priced generics Y: Yes N: No**
