# Supplementary material for: The availability, price and affordability of essential antibacterials in Hubei province, China
Source: BMC Health Serv Res. 2018 Dec 29;18:1013. doi: 10.1186/s12913-018-3835-x (PMC6310993; doi:10.1186/s12913-018-3835-x)
Supplement: Supplementary file 4 — International Ethical Guidelines for Biomedical Research Involving Human Subjects. A guideline of how ethics committee censors research on human subjects. (PDF 166 kb) [file 12913_2018_3835_MOESM4_ESM.pdf]

International Ethical Guidelines for Biomedical Research Involving Human Subjects

Prepared by the Council for International Organizations of Medical Sciences (CIOMS) in collaboration with the World Health Organization (WHO)

CIOMS

Geneva

2002

CONTENTS

◦

ACKNOWLEDGEMENTS

BACKGROUND

INTRODUCTION

INTERNATIONAL INSTRUMENTS AND GUIDELINES

GENERAL ETHICAL PRINCIPLES

PREAMBLE

THE GUIDELINES

Ethical justification and scientific validity of biomedical research involving human subjects

Ethical review

Ethical review committees

Ethical review of externally sponsored research

Informed consent

Individual informed consent

Obtaining informed consent: Essential information for prospective research subjects

Obtaining informed consent: Obligations of sponsors and investigators

Inducement to participate

Benefits and risks of study participation

Special limitations on risk when research involves individuals who are not capable of giving informed consent

* * * * *

Research in populations and communities with limited resources

* * * * *

Choice of control in clinical trials

Vulnerable groups

Equitable distribution of burdens and benefits in the selection of groups of subjects in research

Research involving vulnerable persons

Research involving children

Research involving individuals who by reason of mental or behavioural disorders are not capable of giving adequately informed consent

Women as research participants

Women as research participants

Pregnant women as research participants

* * * * *

Safeguarding confidentiality

Right of injured subjects to treatment and compensation

Strengthening capacity for ethical and scientific review and biomedical research

Ethical obligation of external sponsors to provide health-care services

°

Appendix 1: Items to be included in a protocol (or associated documents) for biomedical research involving human subjects.

Appendix 2: The Declaration of Helsinki

Appendix 3: The phases of clinical trials of vaccines and drugs

ACKNOWLEDGEMENTS

The Council for International Organizations of Medical Sciences (CIOMS) acknowledges the substantial financial contribution of the Joint United Nations Programme on HIV/AIDS (UNAIDS) to the preparation of the 2002 *International Ethical Guidelines for Biomedical Research Involving Human Subjects*. The World Health Organization in Geneva contributed generously also through the departments of Reproductive Health and Research, Essential Drugs and Medicines Policy, Vaccines and Biologicals, and HIV/AIDS/Sexually Transmitted Infections, as well as the Special Programme for Research and Training in Tropical Diseases. CIOMS was at all times free to avail of the services and facilities of WHO.

CIOMS acknowledges also with much appreciation the financial support to the project from the Government of Finland, the Government of Switzerland, the Swiss Academy of Medical Sciences, the Fogarty International Center at the National Institutes of Health, USA, and the Medical Research Council of the United Kingdom.

A number of institutions and organizations made valuable contributions by making their experts available at no cost to CIOMS for the three meetings held in relation to the revision project. This has been highly appreciated.

The task of finalizing the various drafts was in the hands of Professor Robert J. Levine, who served as consultant to the project and chair of the steering committee, and whose profound knowledge and understanding of the field is remarkable. He was ably assisted by Dr James Gallagher of the CIOMS secretariat, who managed the electronic discussion and endeavoured to accommodate or reflect in the text the numerous comments received. He also edited the final text. Special mention must be made of the informal drafting group set up to bring the influence of various cultures to bear on the process. The group, with two members of the CIOMS secretariat, met for five days in New York in January 2001 and continued for several months to interact electronically with one another and with the secretariat to prepare the third draft, posted on the CIOMS website in June 2001: Fernando Lolas Stepke (chair), John Bryant, Leonardo de Castro, Robert Levine, Ruth Macklin, and Godfrey Tangwa; the group continued from October 2001, together with Florencia Luna and Rodolfo Saracci, to cooperate in preparing the fourth draft. The contribution of this group was invaluable.

The interest and comments of the many organizations and individuals who responded to the several drafts of the guidelines posted on the CIOMS website or otherwise made available are gratefully acknowledged (Appendix 6)

At CIOMS, Sev Fluss was at all times ready and resourceful when consulted, with advice and constructive comment, and Mrs Kathryn Chalaby-Amsler responded most competently to the sometimes considerable demands made on her administrative and secretarial skills.

BACKGROUND

The Council for International Organizations of Medical Sciences (CIOMS) is an international nongovernmental organization in official relations with the World Health Organization (WHO). It was founded under the auspices of WHO and the United Nations Educational, Scientific and Cultural and Organization (UNESCO) in 1949 with among its mandates that of maintaining collaborative relations with the United Nations and its specialized agencies, particularly with UNESCO and WHO.

CIOMS, in association with WHO, undertook its work on ethics in relation to biomedical research in the late 1970s. At that time, newly independent WHO Member States were setting up health-care systems. WHO was not then in a position to promote ethics as an aspect of health care or research. It was thus that CIOMS set out, in cooperation with WHO, to prepare guidelines " to indicate how the ethical principles that should guide the conduct of biomedical research involving human subjects, as set forth in the Declaration of Helsinki, could be effectively applied, particularly in developing countries, given their socioeconomic circumstances, laws and regulations, and executive and administrative arrangements". The World Medical Association had issued the original Declaration of Helsinki in 1964 and an amended version in 1975. The outcome of the CIOMS/WHO undertaking was, in 1982, *Proposed International Ethical Guidelines for Biomedical Research Involving Human Subjects*.

The period that followed saw the outbreak of the HIV/AIDS pandemic and proposals to undertake large-scale trials of vaccine and treatment drugs for the condition. These raised new ethical issues that had not been considered in the preparation of *Proposed Guidelines*. There were other factors also – rapid advances in medicine and biotechnology, changing research practices such as multinational field trials, experimentation involving vulnerable population groups, and also a changing view, in rich and poor countries, that research involving human subjects was largely beneficial and not threatening. The Declaration of Helsinki was revised twice in the 1980s – in 1983 and 1989. It was timely to revise and update the 1982 guidelines, and CIOMS, with the cooperation of WHO and its Global Programme on AIDS, undertook the task. The outcome was the issuing of two sets of guidelines: in 1991, *International Guidelines for Ethical Review of Epidemiological Studies*; and, in 1993, *International Ethical Guidelines for Biomedical Research Involving Human Subjects*.

After 1993, ethical issues arose for which the CIOMS Guidelines had no specific provision. They related mainly to controlled clinical trials, with external sponsors and investigators, carried out in low-resource countries and to the use of comparators other than an established effective intervention. The issue in question was the perceived need in those countries for low-cost, technologically appropriate, public-health solutions, and in particular for HIV/AIDS treatment drugs or vaccines that poorer countries could afford. Commentators took opposing sides on this issue. One advocated, for low-resource countries, trials of interventions that, while they might be less effective than the treatment available in the better-off countries, would be less expensive. All research efforts for public solutions appropriate to developing countries should not be rejected as unethical, they claimed. The research context should be considered. Local decision-making should be the norm. Paternalism on the part of the richer countries towards poorer countries should be avoided. The challenge was to encourage research for local solutions to the burden of disease in much of the world, while providing clear guidance on protecting against exploitation of vulnerable communities and individuals.

The other side argued that such trials constituted, or risked constituting, exploitation of poor countries by rich countries and were inherently unethical. Economic factors should not influence ethical considerations. It was within the capacity of rich countries or the pharmaceutical industry to make established effective treatment available for comparator purposes. Certain low-resource countries had already made available from their own resources established effective treatment for their HIV/AIDS patients.

This conflict complicated the revision and updating of the 1993 Guidelines. Ultimately, it became clear that the conflicting views could not be reconciled, though the proponents of the former view claimed that the new guidelines had built in effective safeguards against exploitation. The commentary to the Guideline concerned (11) recognizes the unresolved, or unresolvable, conflict.

The revision/updating of the 1993 Guidelines began in December 1998, and a first draft prepared by the CIOMS consultant for the project was reviewed by the project steering committee, which met in May 1999. The committee proposed amendments and listed topics on which new or revised guidelines were indicated; it recommended papers to be commissioned on the topics, as well as authors and commentators, for presentation and

discussion at a CIOMS interim consultation. It was considered that an interim consultation meeting, of members of the steering committee together with the authors of commissioned papers and designated commentators, followed by further redrafting and electronic distribution and feedback, would better serve the purpose of the project than the process originally envisaged, which had been to complete the revision in one further step. The consultation was accordingly organized for March 2000, in Geneva.

At the consultation, progress on the revision was reported and contentious matters reviewed. Eight commissioned papers previously distributed were presented, commented upon, and discussed. The work of the consultation continued with ad hoc electronic working groups over the following several weeks, and the outcome was made available for the preparation of the third draft. The material commissioned for the consultation was made the subject of a CIOMS publication: *Biomedical Research Ethics: Updating International Guidelines. A Consultation* (December 2000).

An informal redrafting group of eight, from Africa, Asia, Latin America, the United States and the CIOMS secretariat met in New York City in January 2001, and subsequently interacted electronically with one another and with the CIOMS secretariat. A revised draft was posted on the CIOMS website in June 2001 and otherwise widely distributed. Many organizations and individuals commented, some extensively, some critically. Views on certain positions, notably on placebo-controlled trials, were contradictory. For the subsequent revision two members were added to the redrafting group, from Europe and Latin America. The consequent draft was posted on the website in January 2002 in preparation for the CIOMS Conference in February/ March 2002

The CIOMS Conference was convened to discuss and, as far as possible, endorse a final draft to be submitted for final approval to the CIOMS Executive Committee. Besides representation of member organizations of CIOMS, participants included experts in ethics and research from all continents. They reviewed the draft guidelines seriatim and suggested modifications. Guideline 11, *Choice of control in clinical trials*, was redrafted at the conference in an effort to reduce disagreement. The redrafted text of that guideline was intensively discussed and generally well received. Some participants, however, continued to question the ethical acceptability of the exception to the general rule limiting the use of placebo to the conditions set out in the guideline; they argued that research subjects should not be exposed to risk of serious or irreversible harm when an established effective intervention could prevent such harm, and that such exposure could constitute exploitation. Ultimately, the commentary of Guideline 11 reflects the opposing positions on use of a comparator other than an established effective intervention for control purposes.

The new text, the 2002 text, which supersedes that of 1993, consists of a statement of general ethical principles, a preamble and 21 guidelines, with an introduction and a brief account of earlier declarations and guidelines. Like the 1982 and 1993 Guidelines, the present publication is designed to be of use, particularly to low-resource countries, in defining national policies on the ethics of biomedical research, applying ethical standards in local circumstances, and establishing or redefining adequate mechanisms for ethical review of research involving human subjects

Comments on the Guidelines are welcome and should be addressed to the Secretary-General, Council for International Organizations of Medical Sciences, c/o World Health Organization, CH-1211 Geneva 27, Switzerland; or by e-mail to cioms@who.int

INTRODUCTION

This is the third in the series of international ethical guidelines for biomedical research involving human subjects issued by the Council for International Organizations of Medical Sciences since 1982. Its scope and preparation reflect well the transformation that has occurred in the field of research ethics in the almost quarter century since CIOMS first undertook to make this contribution to medical sciences and the ethics of research. The CIOMS Guidelines, with their stated concern for the application of the Declaration of Helsinki in developing countries, necessarily reflect the conditions and the needs of biomedical research in those countries, and the implications for multinational or transnational research in which they may be partners.

An issue, mainly for those countries and perhaps less pertinent now than in the past, has been the extent to which ethical principles are considered universal or as culturally relative – the universalist versus the pluralist view. The challenge to international research ethics is to apply universal ethical principles to biomedical research in a multicultural world with a multiplicity of health-care systems and considerable variation in standards of health care. The Guidelines take the position that research involving human subjects must not violate any universally applicable ethical standards, but acknowledge that, in superficial aspects, the application of the ethical principles, e.g., in relation to individual autonomy and informed consent, needs to take account of cultural values, while respecting absolutely the ethical standards.

Related to this issue is that of the human rights of research subjects, as well as of health professionals as researchers in a variety of sociocultural contexts, and the contribution that international human rights instruments can make in the application of the general principles of ethics to research involving human subjects. The issue concerns largely, though not exclusively, two principles: respect for autonomy and protection of dependent or vulnerable persons and populations. In the preparation of the Guidelines the potential contribution in these respects of human rights instruments and norms was discussed, and the Guideline drafters have represented the views of commentators on safeguarding the corresponding rights of subjects.

Certain areas of research are not represented by specific guidelines. One such is human genetics. It is, however, considered in Guideline 18 Commentary under *Issues of confidentiality in genetics research.* The ethics of genetics research was the subject of a commissioned paper and commentary.

Another unrepresented area is research with products of conception (embryo and fetal research, and fetal tissue research). An attempt to craft a guideline on the topic proved

unfeasible. At issue was the moral status of embryos and fetuses and the degree to which risks to the life or well-being of these entities are ethically permissible.

In relation to the use of comparators in controls, commentators have raised the the question of standard of care to be provided to a control group. They emphasize that standard of care refers to more than the comparator drug or other intervention, and that research subjects in the poorer countries do not usually enjoy the same standard of all-round care enjoyed by subjects in richer countries. This issue is not addressed specifically in the Guidelines.

In one respect the Guidelines depart from the terminology of the Declaration of Helsinki. ‘Best current intervention’ is the term most commonly used to describe the active comparator that is ethically preferred in controlled clinical trials. For many indications, however, there is more than one established ‘current’ intervention and expert clinicians do not agree on which is superior. In other circumstances in which there are several established ‘current’ interventions, some expert clinicians recognize one as superior to the rest; some commonly prescribe another because the superior intervention may be locally unavailable, for example, or prohibitively expensive or unsuited to the capability of particular patients to adhere to a complex and rigorous regimen. ‘Established effective intervention’ is the term used in Guideline 11 to refer to all such interventions, including the best and the various alternatives to the best. In some cases an ethical review committee may determine that it is ethically acceptable to use an established effective intervention as a comparator, even in cases where such an intervention is not considered the best current intervention.

The mere formulation of ethical guidelines for biomedical research involving human subjects will hardly resolve all the moral doubts that can arise in association with much research, but the Guidelines can at least draw the attention of sponsors, investigators and ethical review committees to the need to consider carefully the ethical implications of research protocols and the conduct of research, and thus conduce to high scientific and ethical standards of biomedical research.

INTERNATIONAL INSTRUMENTS AND GUIDELINES

The first international instrument on the ethics of medical research, the Nuremberg Code, was promulgated in 1947 as a consequence of the trial of physicians (the Doctors’ Trial) who had conducted atrocious experiments on unconsenting prisoners and detainees during the second world war. The Code, designed to protect the integrity of the research subject, set out conditions for the ethical conduct of research involving human subjects, emphasizing their voluntary consent to research.

The Universal Declaration of Human Rights was adopted by the General Assembly of the United Nations in 1948. To give the Declaration legal as well as moral force, the General Assembly adopted in 1966 the International Covenant on Civil and Political Rights. Article 7 of the Covenant states "*No one shall be subjected to torture or to cruel,*

inhuman or degrading treatment or punishment. In particular, no one shall be subjected without his free consent to medical or scientific experimentation". It is through this statement that society expresses the fundamental human value that is held to govern all research involving human subjects – the protection of the rights and welfare of all human subjects of scientific experimentation.

The Declaration of Helsinki, issued by the World Medical Association in 1964, is the fundamental document in the field of ethics in biomedical research and has influenced the formulation of international, regional and national legislation and codes of conduct. The Declaration, amended several times, most recently in 2000 (Appendix 2), is a comprehensive international statement of the ethics of research involving human subjects. It sets out ethical guidelines for physicians engaged in both clinical and nonclinical biomedical research.

Since the publication of the CIOMS 1993 Guidelines, several international organizations have issued ethical guidance on clinical trials. This has included, from the World Health Organization, in 1995, *Guidelines for Good Clinical Practice for Trials on Pharmaceutical Products*; and from the International Conference on Harmonisation of Technical Requirements for Registration of Pharmaceuticals for Human Use (ICH), in 1996, *Guideline on Good Clinical Practice*, designed to ensure that data generated from clinical trials are mutually acceptable to regulatory authorities in the European Union, Japan and the United States of America. The Joint United Nations Programme on HIV/AIDS published in 2000 the UNAIDS Guidance Document *Ethical Considerations in HIV Preventive Vaccine Research*.

In 2001 the Council of Ministers of the European Union adopted a Directive on clinical trials, which will be binding in law in the countries of the Union from 2004. The Council of Europe, with more than 40 member States, is developing a Protocol on Biomedical Research, which will be an additional protocol to the Council's 1997 Convention on Human Rights and Biomedicine.

Not specifically concerned with biomedical research involving human subjects but clearly pertinent, as noted above, are international human rights instruments. These are mainly the Universal Declaration of Human Rights, which, particularly in its science provisions, was highly influenced by the Nuremberg Code; the International Covenant on Civil and Political Rights; and the International Covenant on Economic, Social and Cultural Rights. Since the Nuremberg experience, human rights law has expanded to include the protection of women (Convention on the Elimination of All Forms of Discrimination Against Women) and children (Convention on the Rights of the Child). These and other such international instruments endorse in terms of human rights the general ethical principles that underlie the CIOMS International Ethical Guidelines.

GENERAL ETHICAL PRINCIPLES

All research involving human subjects should be conducted in accordance with three basic ethical principles, namely respect for persons, beneficence and justice. It is

generally agreed that these principles, which in the abstract have equal moral force, guide the conscientious preparation of proposals for scientific studies. In varying circumstances they may be expressed differently and given different moral weight, and their application may lead to different decisions or courses of action. The present guidelines are directed at the application of these principles to research involving human subjects.

Respect for persons incorporates at least two fundamental ethical considerations, namely:

> a) respect for autonomy, which requires that those who are capable of deliberation about their personal choices should be treated with respect for their capacity for self-determination; and
>
> b) protection of persons with impaired or diminished autonomy, which requires that those who are dependent or vulnerable be afforded security against harm or abuse.

Beneficence refers to the ethical obligation to maximize benefits and to minimize harms. This principle gives rise to norms requiring that the risks of research be reasonable in the light of the expected benefits, that the research design be sound, and that the investigators be competent both to conduct the research and to safeguard the welfare of the research subjects. Beneficence further proscribes the deliberate infliction of harm on persons; this aspect of beneficence is sometimes expressed as a separate principle, ***nonmaleficence*** (do no harm).

Justice refers to the ethical obligation to treat each person in accordance with what is morally right and proper, to give each person what is due to him or her. In the ethics of research involving human subjects the principle refers primarily to ***distributive justice*,** which requires the equitable distribution of both the burdens and the benefits of participation in research. Differences in distribution of burdens and benefits are justifiable only if they are based on morally relevant distinctions between persons; one such distinction is vulnerability. "Vulnerability" refers to a substantial incapacity to protect one's own interests owing to such impediments as lack of capability to give informed consent, lack of alternative means of obtaining medical care or other expensive necessities, or being a junior or subordinate member of a hierarchical group. Accordingly, special provision must be made for the protection of the rights and welfare of vulnerable persons.

Sponsors of research or investigators cannot, in general, be held accountable for unjust conditions where the research is conducted, but they must refrain from practices that are likely to worsen unjust conditions or contribute to new inequities. Neither should they take advantage of the relative inability of low-resource countries or vulnerable populations to protect their own interests, by conducting research inexpensively and avoiding complex regulatory systems of industrialized countries in order to develop products for the lucrative markets of those countries.

In general, the research project should leave low-resource countries or communities better off than previously or, at least, no worse off. It should be responsive to their health

needs and priorities in that any product developed is made reasonably available to them, and as far as possible leave the population in a better position to obtain effective health care and protect its own health.

Justice requires also that the research be responsive to the health conditions or needs of vulnerable subjects. The subjects selected should be the least vulnerable necessary to accomplish the purposes of the research. Risk to vulnerable subjects is most easily justified when it arises from interventions or procedures that hold out for them the prospect of direct health-related benefit. Risk that does not hold out such prospect must be justified by the anticipated benefit to the population of which the individual research subject is representative.

PREAMBLE

The term "research" refers to a class of activity designed to develop or contribute to generalizable knowledge. Generalizable knowledge consists of theories, principles or relationships, or the accumulation of information on which they are based, that can be corroborated by accepted scientific methods of observation and inference. In the present context "research" includes both medical and behavioural studies pertaining to human health. Usually "research" is modified by the adjective "biomedical" to indicate its relation to health.

Progress in medical care and disease prevention depends upon an understanding of physiological and pathological processes or epidemiological findings, and requires at some time research involving human subjects. The collection, analysis and interpretation of information obtained from research involving human beings contribute significantly to the improvement of human health.

Research involving human subjects includes:

- studies of a physiological, biochemical or pathological process, or of the response to a specific intervention – whether physical, chemical or psychological – in healthy subjects or patients;

- controlled trials of diagnostic, preventive or therapeutic measures in larger groups of persons, designed to demonstrate a specific generalizable response to these measures against a background of individual biological variation;

- studies designed to determine the consequences for individuals and communities of specific preventive or therapeutic measures; and

- studies concerning human health-related behaviour in a variety of circumstances and environments.

Research involving human subjects may employ either observation or physical, chemical or psychological intervention; it may also either generate records or make use of existing records containing biomedical or other information about individuals who may or may not be identifiable from the records or information. The use of such records and the protection of the confidentiality of data obtained from those records are discussed in *International Guidelines for Ethical Review of Epidemiological Studies (CIOMS, 1991).*

The research may be concerned with the social environment, manipulating environmental factors in a way that could affect incidentally-exposed individuals. It is defined in broad terms in order to embrace field studies of pathogenic organisms and toxic chemicals under investigation for health-related purposes.

Biomedical research with human subjects is to be distinguished from the practice of medicine, public health and other forms of health care, which is designed to contribute directly to the health of individuals or communities. Prospective subjects may find it confusing when research and practice are to be conducted simultaneously, as when research is designed to obtain new information about the efficacy of a drug or other therapeutic, diagnostic or preventive modality.

As stated in Paragraph 32 of the Declaration of Helsinki, "In the treatment of a patient, where proven prophylactic, diagnostic and therapeutic methods do not exist or have been ineffective, the physician, with informed consent from the patient, must be free to use unproven or new prophylactic, diagnostic and therapeutic measures, if in the physician's judgement it offers hope of saving life, re-establishing health or alleviating suffering. Where possible, these measures should be made the object of research, designed to evaluate their safety and efficacy. In all cases, new information should be recorded and, where appropriate, published. The other relevant guidelines of this Declaration should be followed."

Professionals whose roles combine investigation and treatment have a special obligation to protect the rights and welfare of the patient-subjects. An investigator who agrees to act as physician-investigator undertakes some or all of the legal and ethical responsibilities of the subject's primary-care physician. In such a case, if the subject withdraws from the research owing to complications related to the research or in the exercise of the right to withdraw without loss of benefit, the physician has an obligation to continue to provide medical care, or to see that the subject receives the necessary care in the health-care system, or to offer assistance in finding another physician.

Research with human subjects should be carried out only by, or strictly supervised by, suitably qualified and experienced investigators and in accordance with a protocol that clearly states: the aim of the research; the reasons for proposing that it involve human subjects; the nature and degree of any known risks to the subjects; the sources from which it is proposed to recruit subjects; and the means proposed for ensuring that subjects' consent will be adequately informed and voluntary. The protocol should be scientifically and ethically appraised by one or more suitably constituted review bodies, independent of the investigators.

New vaccines and medicinal drugs, before being approved for general use, must be tested on human subjects in clinical trials; such trials constitute a substantial part of all research involving human subjects.

THE GUIDELINES

Guideline 1: Ethical justification and scientific validity of biomedical research involving human beings

The ethical justification of biomedical research involving human subjects is the prospect of discovering new ways of benefiting people's health. Such research can be ethically justifiable only if it is carried out in ways that respect and protect, and are fair to, the subjects of that research and are morally acceptable within the communities in which the research is carried out. Moreover, because scientifically invalid research is unethical in that it exposes research subjects to risks without possible benefit, investigators and sponsors must ensure that proposed studies involving human subjects conform to generally accepted scientific principles and are based on adequate knowledge of the pertinent scientific literature.

Commentary on Guideline 1

Among the essential features of ethically justified research involving human subjects, including research with identifiable human tissue or data, are that the research offers a means of developing information not otherwise obtainable, that the design of the research is scientifically sound, and that the investigators and other research personnel are competent. The methods to be used should be appropriate to the objectives of the research and the field of study. Investigators and sponsors must also ensure that all who participate in the conduct of the research are qualified by virtue of their education and experience to perform competently in their roles. These considerations should be adequately reflected in the research protocol submitted for review and clearance to scientific and ethical review committees (Appendix I).

Scientific review is discussed further in the Commentaries to Guidelines 2 and 3: *Ethical review committees* and *Ethical review of externally sponsored research. O*ther ethical aspects of research are discussed in the remaining guidelines and their commentaries. The protocol designed for submission for review and clearance to scientific and ethical review committees should include, when relevant, the items specified in Appendix I, and should be carefully followed in conducting the research.

Guideline 2: Ethical review committees

All proposals to conduct research involving human subjects must be submitted for review of their scientific merit and ethical acceptability to one or more scientific

review and ethical review committees. The review committees must be independent of the research team, and any direct financial or other material benefit they may derive from the research should not be contingent on the outcome of their review. The investigator must obtain their approval or clearance before undertaking the research. The ethical review committee should conduct further reviews as necessary in the course of the research, including monitoring of the progress of the study.

Commentary on Guideline 2

Ethical review committees may function at the institutional, local, regional, or national level, and in some cases at the international level. The regulatory or other governmental authorities concerned should promote uniform standards across committees within a country, and, under all systems, sponsors of research and institutions in which the investigators are employed should allocate sufficient resources to the review process. Ethical review committees may receive money for the activity of reviewing protocols, but under no circumstances may payment be offered or accepted for a review committee`s approval or clearance of a protocol.

Scientific review. According to the Declaration of Helsinki (*Paragraph 11*), medical research involving humans must conform to generally accepted scientific principles, and be based on a thorough knowledge of the scientific literature, other relevant sources of information, and adequate laboratory and, where indicated, animal experimentation. Scientific review must consider, inter alia, the study design, including the provisions for avoiding or minimizing risk and for monitoring safety. Committees competent to review and approve scientific aspects of research proposals must be multidisciplinary.

Ethical review. The ethical review committee is responsible for safeguarding the rights, safety, and well-being of the research subjects. Scientific review and ethical review cannot be separated: scientifically unsound research involving humans as subjects is ipso facto unethical in that it may expose them to risk or inconvenience to no purpose; even if there is no risk of injury, wasting of subjects` and researchers` time in unproductive activities represents loss of a valuable resource. Normally, therefore, an ethical review committee considers both the scientific and the ethical aspects of proposed research. It must either carry out a proper scientific review or verify that a competent expert body has determined that the research is scientifically sound. Also, it considers provisions for monitoring of data and safety.

If the ethical review committee finds a research proposal scientifically sound, or verifies that a competent expert body has found it so, it should then consider whether any known or possible risks to the subjects are justified by the expected benefits, direct or indirect, and whether the proposed research methods will minimize harm and maximize benefit. (See Guideline 8: *Benefits and risks of study participation.)* If the proposal is sound and the balance of risks to anticipated benefits is reasonable, the committee should then determine whether the procedures proposed for obtaining informed consent are satisfactory and those proposed for the selection of subjects are equitable.

Ethical review of emergency compassionate use of an investigational therapy. In some countries, drug regulatory authorities require that the so-called compassionate or

humanitarian use of an investigational treatment be reviewed by an ethical review committee as though it were research. Exceptionally, a physician may undertake the compassionate use of an investigational therapy before obtaining the approval or clearance of an ethical review committee, provided three criteria are met**:** a patient needs emergency treatment, there is some evidence of possible effectiveness of the investigational treatment, and there is no other treatment available that is known to be equally effective or superior. Informed consent should be obtained according to the legal requirements and cultural standards of the community in which the intervention is carried out. Within one week the physician must report to the ethical review committee the details of the case and the action taken, and an independent health-care professional must confirm in writing to the ethical review committee the treating physician's judgment that the use of the investigational intervention was justified according to the three specified criteria. (See also Guideline 13 Commentary section: *Other vulnerable groups.)*

National (centralized) or local review. Ethical review committees may be created under the aegis of national or local health administrations, national (or centralized) medical research councils or other nationally representative bodies. In a highly centralized administration a national, or centralized, review committee may be constituted for both the scientific and the ethical review of research protocols. In countries where medical research is not centrally administered, ethical review is more effectively and conveniently undertaken at a local or regional level. The authority of a local ethical review committee may be confined to a single institution or may extend to all institutions in which biomedical research is carried out within a defined geographical area. The basic responsibilities of ethical review committees are:

- to determine that all proposed interventions, particularly the administration of drugs and vaccines or the use of medical devices or procedures under development, are acceptably safe to be undertaken in humans or to verify that another competent expert body has done so;

- to determine that the proposed research is scientifically sound or to verify that another competent expert body has done so;

- to ensure that all other ethical concerns arising from a protocol are satisfactorily resolved both in principle and in practice;

- to consider the qualifications of the investigators, including education in the principles of research practice, and the conditions of the research site with a view to ensuring the safe conduct of the trial; and

- to keep records of decisions and to take measures to follow up on the conduct of ongoing research projects.

    ○

Committee membership. National or local ethical review committees should be so composed as to be able to provide complete and adequate review of the research

proposals submitted to them. It is generally presumed that their membership should include physicians, scientists and other professionals such as nurses, lawyers, ethicists and clergy, as well as lay persons qualified to represent the cultural and moral values of the community and to ensure that the rights of the research subjects will be respected. They should include both men and women. When uneducated or illiterate persons form the focus of a study they should also be considered for membership or invited to be represented and have their views expressed.

A number of members should be replaced periodically with the aim of blending the advantages of experience with those of fresh perspectives.

A national or local ethical review committee responsible for reviewing and approving proposals for externally sponsored research should have among its members or consultants persons who are thoroughly familiar with the customs and traditions of the population or community concerned and sensitive to issues of human dignity.

Committees that often review research proposals directed at specific diseases or impairments, such as HIV/AIDS or paraplegia, should invite or hear the views of individuals or bodies representing patients with such diseases or impairments. Similarly, for research involving such subjects as children, students, elderly persons or employees, committees should invite or hear the views of their representatives or advocates.

To maintain the review committee's independence from the investigators and sponsors and to avoid conflict of interest, any member with a special or particular, direct or indirect, interest in a proposal should not take part in its assessment if that interest could subvert the member`s objective judgment. Members of ethical review committees should be held to the same standard of disclosure as scientific and medical research staff with regard to financial or other interests that could be construed as conflicts of interest. A practical way of avoiding such conflict of interest is for the committee to insist on a declaration of possible conflict of interest by any of its members. A member who makes such a declaration should then withdraw, if to do so is clearly the appropriate action to take, either at the member`s own discretion or at the request of the other members. Before withdrawing, the member should be permitted to offer comments on the protocol or to respond to questions of other members.

Multi-centre research. Some research projects are designed to be conducted in a number of centres in different communities or countries. Generally, to ensure that the results will be valid, the study must be conducted in an identical way at each centre. Such studies include clinical trials, research designed for the evaluation of health service programmes, and various kinds of epidemiological research. For such studies, local ethical or scientific review committees are not normally authorized to change doses of drugs, to change inclusion or exclusion criteria, or to make other similar modifications. They should be fully empowered to prevent a study that they believe to be unethical. Moreover, changes that local review committees believe are necessary to protect the research subjects should be documented and reported to the research institution or sponsor responsible for the whole research programme for consideration and due action, to ensure that all other subjects can be protected and that the research will be valid across sites.

To ensure the validity of multi-centre research, any change in the protocol should be made at every collaborating centre or institution, or, failing this, explicit inter-centre comparability procedures must be introduced; changes made at some but not all will defeat the purpose of multi-centre research. For some multi-centre studies, scientific and ethical review may be facilitated by agreement among centres to accept the conclusions of a single review committee; its members could include a representative of the ethical review committee at each of the centres at which the research is to be conducted, as well as individuals competent to conduct scientific review. In other circumstances, a centralized review may be complemented by local review relating to the local participating investigators and institutions. The central committee could review the study from a scientific and ethical standpoint, and the local committees could verify the practicability of the study in their communities, including the infrastructures, the state of training, and ethical considerations of local significance.

In a large multi-centre trial, individual investigators will not have authority to act independently, with regard to data analysis or to preparation and publication of manuscripts, for instance. Such a trial usually has a set of committees which operate under the direction of a steering committee and are responsible for such functions and decisions. The function of the ethical review committee in such cases is to review the relevant plans with the aim of avoiding abuses.

Sanctions. Ethical review committees generally have no authority to impose sanctions on researchers who violate ethical standards in the conduct of research involving humans. They may, however, withdraw ethical approval of a research project if judged necessary. They should be required to monitor the implementation of an approved protocol and its progression, and to report to institutional or governmental authorities any serious or continuing non-compliance with ethical standards as they are reflected in protocols that they have approved or in the conduct of the studies. Failure to submit a protocol to the committee should be considered a clear and serious violation of ethical standards.

Sanctions imposed by governmental, institutional, professional or other authorities possessing disciplinary power should be employed as a last resort. Preferred methods of control include cultivation of an atmosphere of mutual trust, and education and support to promote in researchers and in sponsors the capacity for ethical conduct of research.

Should sanctions become necessary, they should be directed at the non-compliant researchers or sponsors. They may include fines or suspension of eligibility to receive research funding, to use investigational interventions, or to practise medicine. Unless there are persuasive reasons to do otherwise, editors should refuse to publish the results of research conducted unethically, and retract any articles that are subsequently found to contain falsified or fabricated data or to have been based on unethical research. Drug regulatory authorities should consider refusal to accept unethically obtained data submitted in support of an application for authorization to market a product. Such sanctions, however, may deprive of benefit not only the errant researcher or sponsor but also that segment of society intended to benefit from the research; such possible consequences merit careful consideration.

Potential conflicts of interest related to project support. Increasingly, biomedical studies receive funding from commercial firms. Such sponsors have good reasons to support research methods that are ethically and scientifically acceptable, but cases have arisen in which the conditions of funding could have introduced bias. It may happen that investigators have little or no input into trial design, limited access to the raw data, or limited participation in data interpretation, or that the results of a clinical trial may not be published if they are unfavourable to the sponsor's product. This risk of bias may also be associated with other sources of support, such as government or foundations. As the persons directly responsible for their work, investigators should not enter into agreements that interfere unduly with their access to the data or their ability to analyse the data independently, to prepare manuscripts, or to publish them. Investigators must also disclose potential or apparent conflicts of interest on their part to the ethical review committee or to other institutional committees designed to evaluate and manage such conflicts. Ethical review committees should therefore ensure that these conditions are met. See also *Multi-centre research*, above.

Guideline 3: Ethical review of externally sponsored research

An external sponsoring organization and individual investigators should submit the research protocol for ethical and scientific review in the country of the sponsoring organization, and the ethical standards applied should be no less stringent than they would be for research carried out in that country. The health authorities of the host country, as well as a national or local ethical review committee, should ensure that the proposed research is responsive to the health needs and priorities of the host country and meets the requisite ethical standards.

Commentary on Guideline 3

Definition. The term *externally sponsored research* refers to research undertaken in a host country but sponsored, financed, and sometimes wholly or partly carried out by an external international or national organization or pharmaceutical company with the collaboration or agreement of the appropriate authorities, institutions and personnel of the host country.

Ethical and scientific review. Committees in both the country of the sponsor and the host country have responsibility for conducting both scientific and ethical review, as well as the authority to withhold approval of research proposals that fail to meet their scientific or ethical standards. As far as possible, there must be assurance that the review is independent and that there is no conflict of interest that might affect the judgement of members of the review committees in relation to any aspect of the research. When the external sponsor is an international organization, its review of the research protocol must be in accordance with its own independent ethical-review procedures and standards.

Committees in the external sponsoring country or international organization have a special responsibility to determine whether the scientific methods are sound and suitable to the aims of the research; whether the drugs, vaccines, devices or procedures to be

studied meet adequate standards of safety; whether there is sound justification for conducting the research in the host country rather than in the country of the external sponsor or in another country; and whether the proposed research is in compliance with the ethical standards of the external sponsoring country or international organization.

Committees in the host country have a special responsibility to determine whether the objectives of the research are responsive to the health needs and priorities of that country. The ability to judge the ethical acceptability of various aspects of a research proposal requires a thorough understanding of a community's customs and traditions. The ethical review committee in the host country, therefore, must have as either members or consultants persons with such understanding; it will then be in a favourable position to determine the acceptability of the proposed means of obtaining informed consent and otherwise respecting the rights of prospective subjects as well as of the means proposed to protect the welfare of the research subjects. Such persons should be able, for example, to indicate suitable members of the community to serve as intermediaries between investigators and subjects, and to advise on whether material benefits or inducements may be regarded as appropriate in the light of a community's gift-exchange and other customs and traditions.

When a sponsor or investigator in one country proposes to carry out research in another, the ethical review committees in the two countries may, by agreement, undertake to review different aspects of the research protocol. In short, in respect of host countries either with developed capacity for independent ethical review or in which external sponsors and investigators are contributing substantially to such capacity, ethical review in the external, sponsoring country may be limited to ensuring compliance with broadly stated ethical standards. The ethical review committee in the host country can be expected to have greater competence for reviewing the detailed plans for compliance, in view of its better understanding of the cultural and moral values of the population in which it is proposed to conduct the research; it is also likely to be in a better position to monitor compliance in the course of a study. However, in respect of research in host countries with inadequate capacity for independent ethical review, full review by the ethical review committee in the external sponsoring country or international agency is necessary.

Guideline 4: Individual informed consent

For all biomedical research involving humans the investigator must obtain the voluntary informed consent of the prospective subject or, in the case of an individual who is not capable of giving informed consent, the permission of a legally authorized representative in accordance with applicable law. Waiver of informed consent is to be regarded as uncommon and exceptional, and must in all cases be approved by an ethical review committee.

Commentary on Guideline 4

General considerations. Informed consent is a decision to participate in research, taken by a competent individual who has received the necessary information; who has adequately understood the information; and who, after considering the information, has arrived at a decision without having been subjected to coercion, undue influence or inducement, or intimidation.

Informed consent is based on the principle that competent individuals are entitled to choose freely whether to participate in research. Informed consent protects the individual's freedom of choice and respects the individual's autonomy. As an additional safeguard, it must always be complemented by independent ethical review of research proposals. This safeguard of independent review is particularly important as many individuals are limited in their capacity to give adequate informed consent; they include young children, adults with severe mental or behavioural disorders, and persons who are unfamiliar with medical concepts and technology (See Guidelines 13, 14, 15).

Process. Obtaining informed consent is a process that is begun when initial contact is made with a prospective subject and continues throughout the course of the study. By informing the prospective subjects, by repetition and explanation, by answering their questions as they arise, and by ensuring that each individual understands each procedure, investigators elicit their informed consent and in so doing manifest respect for their dignity and autonomy. Each individual must be given as much time as is needed to reach a decision, including time for consultation with family members or others. Adequate time and resources should be set aside for informed-consent procedures.

Language. Informing the individual subject must not be simply a ritual recitation of the contents of a written document. Rather, the investigator must convey the information, whether orally or in writing, in language that suits the individual's level of understanding. The investigator must bear in mind that the prospective subject`s ability to understand the information necessary to give informed consent depends on that individual's maturity, intelligence, education and belief system. It depends also on the investigator's ability and willingness to communicate with patience and sensitivity.

Comprehension. The investigator must then ensure that the prospective subject has adequately understood the information. The investigator should give each one full opportunity to ask questions and should answer them honestly, promptly and completely. In some instances the investigator may administer an oral or a written test or otherwise determine whether the information has been adequately understood.

Documentation of consent. Consent may be indicated in a number of ways. The subject may imply consent by voluntary actions, express consent orally, or sign a consent form. As a general rule, the subject should sign a consent form, or, in the case of incompetence, a legal guardian or other duly authorized representative should do so. The ethical review committee may approve waiver of the requirement of a signed consent form if the research carries no more than minimal risk – that is, risk that is no more likely and not greater than that attached to routine medical or psychological examination – and if the procedures to be used are only those for which signed consent forms are not customarily required outside the research context. Such waivers may also be approved when existence of a signed consent form would be an unjustified threat to the subject's confidentiality. In

some cases, particularly when the information is complicated, it is advisable to give subjects information sheets to retain; these may resemble consent forms in all respects except that subjects are not required to sign them. Their wording should be cleared by the ethical review committee. When consent has been obtained orally, investigators are responsible for providing documentation or proof of consent.

Waiver of the consent requirement. Investigators should never initiate research involving human subjects without obtaining each subject's informed consent, unless they have received explicit approval to do so from an ethical review committee. However, when the research design involves no more than minimal risk and a requirement of individual informed consent would make the conduct of the research impracticable (for example, where the research involves only excerpting data from subjects' records), the ethical review committee may waive some or all of the elements of informed consent.

Renewing consent. When material changes occur in the conditions or the procedures of a study, and also periodically in long-term studies, the investigator should once again seek informed consent from the subjects. For example, new information may have come to light, either from the study or from other sources, about the risks or benefits of products being tested or about alternatives to them. Subjects should be given such information promptly. In many clinical trials, results are not disclosed to subjects and investigators until the study is concluded. This is ethically acceptable if an ethical review committee has approved their non-disclosure.

Cultural considerations. In some cultures an investigator may enter a community to conduct research or approach prospective subjects for their individual consent only after obtaining permission from a community leader, a council of elders, or another designated authority. Such customs must be respected. In no case, however, may the permission of a community leader or other authority substitute for individual informed consent. In some populations the use of a number of local languages may complicate the communication of information to potential subjects and the ability of an investigator to ensure that they truly understand it. Many people in all cultures are unfamiliar with, or do not readily understand, scientific concepts such as those of placebo or randomization. Sponsors and investigators should develop culturally appropriate ways to communicate information that is necessary for adherence to the standard required in the informed consent process. Also, they should describe and justify in the research protocol the procedure they plan to use in communicating information to subjects. For collaborative research in developing countries the research project should, if necessary, include the provision of resources to ensure that informed consent can indeed be obtained legitimately within different linguistic and cultural settings.

Consent to use for research purposes biological materials (including genetic material) from subjects in clinical trials. Consent forms for the research protocol should include a separate section for clinical-trial subjects who are requested to provide their consent for the use of their biological specimens for research. Separate consent may be appropriate in some cases (e.g., if investigators are requesting permission to conduct basic research which is not a necessary part of the clinical trial), but not in others (e.g., the clinical trial requires the use of subjects’ biological materials).

Use of medical records and biological specimens. Medical records and biological specimens taken in the course of clinical care may be used for research without the consent of the patients/subjects only if an ethical review committee has determined that the research poses minimal risk, that the rights or interests of the patients will not be violated, that their privacy and confidentiality or anonymity are assured, and that the research is designed to answer an important question and would be impracticable if the requirement for informed consent were to be imposed. Patients have a right to know that their records or specimens may be used for research. Refusal or reluctance of individuals to agree to participate would not be evidence of impracticability sufficient to warrant waiving informed consent. Records and specimens of individuals who have specifically rejected such uses in the past may be used only in the case of public health emergencies**.** (See Guideline 18 Commentary, *Confidentiality between physician and patient)*

Secondary use of research records or biological specimens. Investigators may want to use records or biological specimens that another investigator has used or collected for use, in another institution in the same or another country. This raises the issue of whether the records or specimens contain personal identifiers, or can be linked to such identifiers, and by whom. (See also Guideline 18: *Safeguarding confidentiality*) If informed consent or permission was required to authorize the original collection or use of such records or specimens for research purposes, secondary uses are generally constrained by the conditions specified in the original consent. Consequently, it is essential that the original consent process anticipate, to the extent that this is feasible, any foreseeable plans for future use of the records or specimens for research. Thus, in the original process of seeking informed consent a member of the research team should discuss with, and, when indicated, request the permission of, prospective subjects as to: i) whether there will or could be any secondary use and, if so, whether such secondary use will be limited with regard to the type of study that may be performed on such materials; ii) the conditions under which investigators will be required to contact the research subjects for additional authorization for secondary use; iii) the investigators' plans, if any, to destroy or to strip of personal identifiers the records or specimens; and iv) the rights of subjects to request destruction or anonymization of biological specimens or of records or parts of records that they might consider particularly sensitive, such as photographs, videotapes or audiotapes.

(See also Guidelines 5: *Obtaining informed consent: Essential information for prospective research subjects;* 6: *Obtaining informed consent: Obligations of sponsors and investigators;* and 7: *Inducement to participate.)*

Guideline 5: Obtaining informed consent: Essential information for prospective research subjects

Before requesting an individual's consent to participate in research, the investigator must provide the following information, in language or another form of communication that the individual can understand:

1. **that the individual is invited to participate in research, the reasons for considering the individual suitable for the research, and that participation is voluntary;**

2. **that the individual is free to refuse to participate and will be free to withdraw from the research at any time without penalty or loss of benefits to which he or she would otherwise be entitled;**

3. **the purpose of the research, the procedures to be carried out by the investigator and the subject, and an explanation of how the research differs from routine medical care;**

4. **for controlled trials, an explanation of features of the research design (e.g., randomization, double-blinding), and that the subject will not be told of the assigned treatment until the study has been completed and the blind has been broken;**

5. **the expected duration of the individual's participation (including number and duration of visits to the research centre and the total time involved) and the possibility of early termination of the trial or of the individual s participation in it;**

6. **whether money or other forms of material goods will be provided in return for the individual's participation and, if so, the kind and amount;**

7. **that, after the completion of the study, subjects will be informed of the findings of the research in general, and individual subjects will be informed of any finding that relates to their particular health status;**

8. **that subjects have the right of access to their data on demand, even if these data lack immediate clinical utility (unless the ethical review committee has approved temporary or permanent non-disclosure of data, in which case the subject should be informed of, and given, the reasons for such non-disclosure);**

9. **any foreseeable risks, pain or discomfort, or inconvenience to the individual (or others) associated with participation in the research, including risks to the health or well-being of a subject s spouse or partner;**

10. **the direct benefits, if any, expected to result to subjects from participating in the research**

11. **the expected benefits of the research to the community or to society at large, or contributions to scientific knowledge;**

12. **whether, when and how any products or interventions proven by the research to be safe and effective will be made available to subjects after they have completed their participation in the research, and whether they will be expected to pay for them;**

13. **any currently available alternative interventions or courses of treatment;**

14. **the provisions that will be made to ensure respect for the privacy of subjects and for the confidentiality of records in which subjects are identified;**

15. **the limits, legal or other, to the investigators' ability to safeguard confidentiality, and the possible consequences of breaches of confidentiality;**

16. **policy with regard to the use of results of genetic tests and familial genetic information, and the precautions in place to prevent disclosure of the results of a subject's genetic tests**

17. **to immediate family relatives or to others (e.g., insurance companies or employers) without the consent of the subject;**

18. **the sponsors of the research, the institutional affiliation of the investigators, and the nature and sources of funding for the research;**

19. **the possible research uses, direct or secondary, of the subject's medical records and of biological specimens taken in the course of clinical care (See also Guidelines 4 and 18 Commentaries);**

20. **whether it is planned that biological specimens collected in the research will be destroyed at its conclusion, and, if not, details about their storage (where, how, for how long, and final disposition) and possible future use, and that subjects have the right to decide about such future use, to refuse storage, and to have the material destroyed (See Guideline 4 Commentary);**

21. **whether commercial products may be developed from biological specimens, and whether the participant will receive monetary or other benefits from the development of such products;**

22. **whether the investigator is serving only as an investigator or as both investigator and the subject's physician;**

23. **the extent of the investigator's responsibility to provide medical services to the participant;**

24. **that treatment will be provided free of charge for specified types of research-related injury or for complications associated with the research, the nature and duration of such care, the name of the organization or individual that will provide the treatment, and whether there is any uncertainty regarding funding of such treatment.**

25. **in what way, and by what organization, the subject or the subject's family or dependants will be compensated for disability or death resulting from such injury (or, when indicated, that there are no plans to provide such compensation);**

26. **whether or not, in the country in which the prospective subject is invited to participate in research, the right to compensation is legally guaranteed;**

27. **that an ethical review committee has approved or cleared the research protocol.**

Guideline 6: Obtaining informed consent: Obligations of sponsors and investigators

Sponsors and investigators have a duty to:

- **refrain from unjustified deception, undue influence, or intimidation;**

- **seek consent only after ascertaining that the prospective subject has adequate understanding of the relevant facts and of the consequences of participation and has had sufficient opportunity to consider whether to participate;**

- **as a general rule, obtain from each prospective subject a signed form as evidence of informed consent — investigators should justify any exceptions to this general rule and obtain the approval of the ethical review committee (See Guideline 4 Commentary, *Documentation of consent*);**

- **renew the informed consent of each subject if there are significant changes in the conditions or procedures of the research or if new information becomes available that could affect the willingness of subjects to continue to participate; and,**

- **renew the informed consent of each subject in long-term studies at pre-determined intervals, even if there are no changes in the design or objectives of the research.**

Commentary on Guideline 6

The investigator is responsible for ensuring the adequacy of informed consent from each subject. The person obtaining informed consent should be knowledgeable about the research and capable of answering questions from prospective subjects. Investigators in charge of the study must make themselves available to answer questions at the request of subjects. Any restrictions on the subject`s opportunity to ask questions and receive answers before or during the research undermines the validity of the informed consent.

In some types of research, potential subjects should receive counselling about risks of acquiring a disease unless they take precautions. This is especially true of HIV/AIDS vaccine research (UNAIDS Guidance Document *Ethical Considerations in HIV Preventive Vaccine Research, Guidance Point 14).*

Withholding information and deception. Sometimes, to ensure the validity of research, investigators withhold certain information in the consent process. In biomedical research, this typically takes the form of withholding information about the purpose of specific procedures. For example, subjects in clinical trials are often not told the purpose of tests performed to monitor their compliance with the protocol, since if they knew their compliance was being monitored they might modify their behaviour and hence invalidate results. In most such cases, the prospective subjects are asked to consent to remain uninformed of the purpose of some procedures until the research is completed; after the conclusion of the study they are given the omitted information. In other cases, because a request for permission to withhold some information would jeopardize the validity of the research, subjects are not told that some information has been withheld until the research has been completed. Any such procedure must receive the explicit approval of the ethical review committee.

Active deception of subjects is considerably more controversial than simply withholding certain information. Lying to subjects is a tactic not commonly employed in biomedical research. Social and behavioural scientists, however, sometimes deliberately misinform subjects to study their attitudes and behaviour. For example, scientists have pretended to be patients to study the behaviour of health-care professionals and patients in their natural settings.

Some people maintain that active deception is never permissible. Others would permit it in certain circumstances. Deception is not permissible, however, in cases in which the deception itself would disguise the possibility of the subject being exposed to more than minimal risk. When deception is deemed indispensable to the methods of a study the investigators must demonstrate to an ethical review committee that no other research method would suffice; that significant advances could result from the research; and that nothing has been withheld that, if divulged, would cause a reasonable person to refuse to participate. The ethical review committee should determine the consequences for the subject of being deceived, and whether and how deceived subjects should be informed of the deception upon completion of the research. Such informing, commonly called "debriefing", ordinarily entails explaining the reasons for the deception. A subject who disapproves of having been deceived should be offered an opportunity to refuse to allow the investigator to use information thus obtained. Investigators and ethical review

committees should be aware that deceiving research subjects may wrong them as well as harm them; subjects may resent not having been informed when they learn that they have participated in a study under false pretences. In some studies there may be justification for deceiving persons other than the subjects by either withholding or disguising elements of information. Such tactics are often proposed, for example, for studies of the abuse of spouses or children. An ethical review committee must review and approve all proposals to deceive persons other than the subjects. Subjects are entitled to prompt and honest answers to their questions; the ethical review committee must determine for each study whether others who are to be deceived are similarly entitled.

Intimidation and undue influence. Intimidation in any form invalidates informed consent. Prospective subjects who are patients often depend for medical care upon the physician/investigator, who consequently has a certain credibility in their eyes, and whose influence over them may be considerable, particularly if the study protocol has a therapeutic component**.** They may fear, for example, that refusal to participate would damage the therapeutic relationship or result in the withholding of health services. The physician/investigator must assure them that their decision on whether to participate will not affect the therapeutic relationship or other benefits to which they are entitled. In this situation the ethical review committee should consider whether a neutral third party should seek informed consent.

The prospective subject must not be exposed to undue influence. The borderline between justifiable persuasion and undue influence is imprecise, however. The researcher should give no unjustifiable assurances about the benefits, risks or inconveniences of the research, for example, or induce a close relative or a community leader to influence a prospective subject's decision. (See also Guideline 4: *Individual informed consent.)*

Risks. Investigators should be completely objective in discussing the details of the experimental intervention, the pain and discomfort that it may entail, and known risks and possible hazards. In complex research projects it may be neither feasible nor desirable to inform prospective participants fully about every possible risk. They must, however, be informed of all risks that a ‘reasonable person’ would consider material to making a decision about whether to participate, including risks to a spouse or partner associated with trials of, for example, psychotropic or genital-tract medicaments. (See also Guideline 8 Commentary, *Risks to groups of persons.*)

Exception to the requirement for informed consent in studies of emergency situations in which the researcher anticipates that many subjects will be unable to consent. Research protocols are sometimes designed to address conditions occurring suddenly and rendering the patients/subjects incapable of giving informed consent. Examples are head trauma, cardiopulmonary arrest and stroke. The investigation cannot be done with patients who can give informed consent in time and there may not be time to locate a person having the authority to give permission. In such circumstances it is often necessary to proceed with the research interventions very soon after the onset of the condition in order to evaluate an investigational treatment or develop the desired knowledge. As this class of emergency exception can be anticipated, the researcher must secure the review and approval of an ethical review committee before initiating the study. If possible, an attempt should be made to identify a population that is likely to develop the condition to

be studied. This can be done readily, for example, if the condition is one that recurs periodically in individuals; examples include grand mal seizures and alcohol binges. In such cases, prospective subjects should be contacted while fully capable of informed consent, and invited to consent to their involvement as research subjects during future periods of incapacitation. If they are patients of an independent physician who is also the physician-researcher, the physician should likewise seek their consent while they are fully capable of informed consent. In all cases in which approved research has begun without prior consent of patients/subjects incapable of giving informed consent because of suddenly occurring conditions, they should be given all relevant information as soon as they are in a state to receive it, and their consent to continued participation should be obtained as soon as is reasonably possible**.**

Before proceeding without prior informed consent, the investigator must make reasonable efforts to locate an individual who has the authority to give permission on behalf of an incapacitated patient. If such a person can be located and refuses to give permission, the patient may not be enrolled as a subject**.** The risks of all interventions and procedures will be justified as required by Guideline 9 (*Special limitations on risks when research involves individuals who are not capable of giving consent).* The researcher and the ethical review committee should agree to a maximum time of involvement of an individual without obtaining either the individual's informed consent or authorization according to the applicable legal system if the person is not able to give consent. If by that time the researcher has not obtained either consent or permission – owing either to a failure to contact a representative or to a refusal of either the patient or the person or body authorized to give permission – the participation of the patient as a subject must be discontinued. The patient or the person or body providing authorization should be offered an opportunity to forbid the use of data derived from participation of the patient as a subject without consent or permission.

Where appropriate, plans to conduct emergency research without prior consent of the subjects should be publicized within the community in which it will be carried out. In the design and conduct of the research, the ethical review committee, the investigators and the sponsors should be responsive to the concerns of the community. If there is cause for concern about the acceptability of the research in the community, there should be a formal consultation with representatives designated by the community. The research should not be carried out if it does not have substantial support in the community concerned. (See Guideline 8 Commentary, *Risks to groups of persons*.)

Exception to the requirement of informed consent for inclusion in clinical trials of persons rendered incapable of informed consent by an acute condition. Certain patients with an acute condition that renders them incapable of giving informed consent may be eligible for inclusion in a clinical trial in which the majority of prospective subjects will be capable of informed consent. Such a trial would relate to a new treatment for an acute condition such as sepsis, stroke or myocardial infarction. The investigational treatment would hold out the prospect of direct benefit and would be justified accordingly, though the investigation might involve certain procedures or interventions that were not of direct benefit but carried no more than minimal risk; an example would be the process of randomization or the collection of additional blood for research purposes. For such cases

the initial protocol submitted for approval to the ethical review committee should anticipate that some patients may be incapable of consent, and should propose for such patients a form of proxy consent, such as permission of the responsible relative. When the ethical review committee has approved or cleared such a protocol, an investigator may seek the permission of the responsible relative and enrol such a patient.

Guideline 7: Inducement to participate

Subjects may be reimbursed for lost earnings, travel costs and other expenses incurred in taking part in a study; they may also receive free medical services. Subjects, particularly those who receive no direct benefit from research, may also be paid or otherwise compensated for inconvenience and time spent. The payments should not be so large, however, or the medical services so extensive as to induce prospective subjects to consent to participate in the research against their better judgment ("undue inducement"). All payments, reimbursements and medical services provided to research subjects must have been approved by an ethical review committee.

Commentary on Guideline 7

Acceptable recompense. Research subjects may be reimbursed for their transport and other expenses, including lost earnings, associated with their participation in research. Those who receive no direct benefit from the research may also receive a small sum of money for inconvenience due to their participation in the research. All subjects may receive medical services unrelated to the research and have procedures and tests performed free of charge.

Unacceptable recompense. Payments in money or in kind to research subjects should not be so large as to persuade them to take undue risks or volunteer against their better judgment. Payments or rewards that undermine a person's capacity to exercise free choice invalidate consent. It may be difficult to distinguish between suitable recompense and undue influence to participate in research. An unemployed person or a student may view promised recompense differently from an employed person. Someone without access to medical care may or may not be unduly influenced to participate in research simply to receive such care. A prospective subject may be induced to participate in order to obtain a better diagnosis or access to a drug not otherwise available; local ethical review committees may find such inducements acceptable. Monetary and in-kind recompense must, therefore, be evaluated in the light of the traditions of the particular culture and population in which they are offered, to determine whether they constitute undue influence. The ethical review committee will ordinarily be the best judge of what constitutes reasonable material recompense in particular circumstances. When research interventions or procedures that do not hold out the prospect of direct benefit present more than minimal risk, all parties involved in the research – sponsors, investigators and

ethical review committees – in both funding and host countries should be careful to avoid undue material inducement.

Incompetent persons. Incompetent persons may be vulnerable to exploitation for financial gain by guardians. A guardian asked to give permission on behalf of an incompetent person should be offered no recompense other than a refund of travel and related expenses.

Withdrawal from a study. A subject who withdraws from research for reasons related to the study, such as unacceptable side-effects of a study drug, or who is withdrawn on health grounds, should be paid or recompensed as if full participation had taken place. A subject who withdraws for any other reason should be paid in proportion to the amount of participation. An investigator who must remove a subject from the study for wilful noncompliance is entitled to withhold part or all of the payment.

°

Guideline 8: Benefits and risks of study participation

For all biomedical research involving human subjects, the investigator must ensure that potential benefits and risks are reasonably balanced and risks are minimized.

- **Interventions or procedures that hold out the prospect of direct diagnostic, therapeutic or preventive benefit for the individual subject must be justified by the expectation that they will be at least as advantageous to the individual subject, in the light of foreseeable risks and benefits, as any available alternative. Risks of such 'beneficial' interventions or procedures must be justified in relation to expected benefits to the individual subject.**

- **Risks of interventions that do not hold out the prospect of direct diagnostic, therapeutic or preventive benefit for the individual must be justified in relation to the expected benefits to society (generalizable knowledge). The risks presented by such interventions must be reasonable in relation to the importance of the knowledge to be gained.**

Commentary on Guideline 8

The Declaration of Helsinki in several paragraphs deals with the well-being of research subjects and the avoidance of risk. Thus, considerations related to the well-being of the human subject should take precedence over the interests of science and society *(Paragraph 5*); clinical testing must be preceded by adequate laboratory or animal experimentation to demonstrate a reasonable probability of success without undue risk (*Paragraph 11*); every project should be preceded by careful assessment of predictable risks and burdens in comparison with foreseeable benefits to the subject or to others (*Paragraph 16*); physician-researchers must be confident that the risks involved have been adequately assessed and can be satisfactorily managed (*Paragraph 17*); and the

risks and burdens to the subject must be minimized, and reasonable in relation to the importance of the objective or the knowledge to be gained (*Paragraph 18*).

Biomedical research often employs a variety of interventions of which some hold out the prospect of direct therapeutic benefit (beneficial interventions) and others are administered solely to answer the research question (non-beneficial interventions). Beneficial interventions are justified as they are in medical practice by the expectation that they will be at least as advantageous to the individuals concerned, in the light of both risks and benefits, as any available alternative. Non-beneficial interventions are assessed differently; they may be justified only by appeal to the knowledge to be gained. In assessing the risks and benefits that a protocol presents to a population, it is appropriate to consider the harm that could result from forgoing the research.

Paragraphs 5 and 18 of the Declaration of Helsinki do not preclude well-informed volunteers, capable of fully appreciating risks and benefits of an investigation, from participating in research for altruistic reasons or for modest remuneration.

Minimizing risk associated with participation in a randomized controlled trial. In randomized controlled trials subjects risk being allocated to receive the treatment that proves inferior. They are allocated by chance to one of two or more intervention arms and followed to a predetermined end-point. (Interventions are understood to include new or established therapies, diagnostic tests and preventive measures.) An intervention is evaluated by comparing it with another intervention (a control), which is ordinarily the best current method, selected from the safe and effective treatments available globally, unless some other control intervention such as placebo can be justified ethically (See Guideline 11).

To minimize risk when the intervention to be tested in a randomized controlled trial is designed to prevent or postpone a lethal or disabling outcome, the investigator must not, for purposes of conducting the trial, withhold therapy that is known to be superior to the intervention being tested, unless the withholding can be justified by the standards set forth in Guideline 11. Also, the investigator must provide in the research protocol for the monitoring of research data by an independent board (Data and Safety Monitoring Board); one function of such a board is to protect the research subjects from previously unknown adverse reactions or unnecessarily prolonged exposure to an inferior therapy. Normally at the outset of a randomized controlled trial, criteria are established for its premature termination (stopping rules or guidelines).

Risks to groups of persons. Research in certain fields, such as epidemiology, genetics or sociology, may present risks to the interests of communities, societies, or racially or ethnically defined groups. Information might be published that could stigmatize a group or expose its members to discrimination. Such information, for example, could indicate, rightly or wrongly, that the group has a higher than average prevalence of alcoholism, mental illness or sexually transmitted disease, or is particularly susceptible to certain genetic disorders. Plans to conduct such research should be sensitive to such considerations, to the need to maintain confidentiality during and after the study, and to the need to publish the resulting data in a manner that is respectful of the interests of all concerned, or in certain circumstances not to publish them. The ethical review committee

should ensure that the interests of all concerned are given due consideration; often it will be advisable to have individual consent supplemented by community consultation.

[The ethical basis for the justification of risk is elaborated further in Guideline 9]

***Guideline 9*: *Special limitations on risk when research involves* individuals who are not capable of giving informed consent**

When there is ethical and scientific justification to conduct research with individuals incapable of giving informed consent, the risk from research interventions that do not hold out the prospect of direct benefit for the individual subject should be no more likely and not greater than the risk attached to routine medical or psychological examination of such persons. Slight or minor increases above such risk may be permitted when there is an overriding scientific or medical rationale for such increases and when an ethical review committee has approved them.

Commentary on Guideline 9

The low-risk standard: Certain individuals or groups may have limited capacity to give informed consent either because, as in the case of prisoners, their autonomy is limited, or because they have limited cognitive capacity. For research involving persons who are unable to consent, or whose capacity to make an informed choice may not fully meet the standard of informed consent, ethical review committees must distinguish between intervention risks that do not exceed those associated with routine medical or psychological examination of such persons and risks in excess of those.

When the risks of such interventions do not exceed those associated with routine medical or psychological examination of such persons, there is no requirement for special substantive or procedural protective measures apart from those generally required for all research involving members of the particular class of persons. When the risks are in excess of those, the ethical review committee must find: 1) that the research is designed to be responsive to the disease affecting the prospective subjects or to conditions to which they are particularly susceptible; 2) that the risks of the research interventions are only slightly greater than those associated with routine medical or psychological examination of such persons for the condition or set of clinical circumstances under investigation; 3) that the objective of the research is sufficiently important to justify exposure of the subjects to the increased risk; and 4) that the interventions are reasonably commensurate with the clinical interventions that the subjects have experienced or may be expected to experience in relation to the condition under investigation.

If such research subjects, including children, become capable of giving independent informed consent during the research, their consent to continued participation should be obtained.

There is no internationally agreed, precise definition of a "slight or minor increase" above the risks associated with routine medical or psychological examination of such persons. Its meaning is inferred from what various ethical review committees have reported as having met the standard. Examples include additional lumbar punctures or bone-marrow aspirations in children with conditions for which such examinations are regularly indicated in clinical practice. The requirement that the objective of the research be relevant to the disease or condition affecting the prospective subjects rules out the use of such interventions in healthy children.

The requirement that the research interventions be reasonably commensurate with clinical interventions that subjects may have experienced or are likely to experience for the condition under investigation is intended to enable them to draw on personal experience as they decide whether to accept or reject additional procedures for research purposes. Their choices will, therefore, be more informed even though they may not fully meet the standard of informed consent.

(See also Guidelines 4: *Individual informed consent*; 13: *Research involving vulnerable persons;* 14: *Research involving children;* and 15: *Research involving individuals who by reason of mental or behavioural disorders are not capable of giving adequately informed consent.)*

◦

Guideline 10: Research in populations and communities with limited resources

Before undertaking research in a population or community with limited resources, the sponsor and the investigator must make every effort to ensure that:

- **the research is responsive to the health needs and the priorities of the population or community in which it is to be carried out; and**

- **any intervention or product developed, or knowledge generated, will be made reasonably available for the benefit of that population or community.**

Commentary on Guideline 10

This guideline is concerned with countries or communities in which resources are limited to the extent that they are, or may be, vulnerable to exploitation by sponsors and investigators from the relatively wealthy countries and communities.

Responsiveness of research to health needs and priorities. The ethical requirement that research be responsive to the health needs of the population or community in which it is carried out calls for decisions on what is needed to fulfil the requirement**.** It is not sufficient simply to determine that a disease is prevalent in the population and that new or further research is needed: the ethical requirement of "responsiveness" can be fulfilled only if successful interventions or other kinds of health benefit are made available to the

population. This is applicable especially to research conducted in countries where governments lack the resources to make such products or benefits widely available. Even when a product to be tested in a particular country is much cheaper than the standard treatment in some other countries, the government or individuals in that country may still be unable to afford it. If the knowledge gained from the research in such a country is used primarily for the benefit of populations that can afford the tested product, the research may rightly be characterized as exploitative and, therefore, unethical.

When an investigational intervention has important potential for health care in the host country, the negotiation that the sponsor should undertake to determine the practical implications of "responsiveness", as well as "reasonable availability", should include representatives of stakeholders in the host country; these include the national government, the health ministry, local health authorities, and concerned scientific and ethics groups, as well as representatives of the communities from which subjects are drawn and non-governmental organizations such as health advocacy groups. The negotiation should cover the health-care infrastructure required for safe and rational use of the intervention, the likelihood of authorization for distribution, and decisions regarding payments, royalties, subsidies, technology and intellectual property, as well as distribution costs, when this economic information is not proprietary. In some cases, satisfactory discussion of the availability and distribution of successful products will necessarily engage international organizations, donor governments and bilateral agencies, international nongovernmental organizations, and the private sector. The development of a health-care infrastructure should be facilitated at the onset so that it can be of use during and beyond the conduct of the research.

Additionally, if an investigational drug has been shown to be beneficial, the sponsor should continue to provide it to the subjects after the conclusion of the study, and pending its approval by a drug regulatory authority. The sponsor is unlikely to be in a position to make a beneficial investigational intervention generally available to the community or population until some time after the conclusion of the study, as it may be in short supply and in any case cannot be made generally available before a drug regulatory authority has approved it.

For minor research studies and when the outcome is scientific knowledge rather than a commercial product, such complex planning or negotiation is rarely, if ever, needed. There must be assurance, however, that the scientific knowledge developed will be used for the benefit of the population.

Reasonable availability. The issue of "reasonable availability" is complex and will need to be determined on a case-by-case basis. Relevant considerations include the length of time for which the intervention or product developed, or other agreed benefit, will be made available to research subjects, or to the community or population concerned; the severity of a subject's medical condition; the effect of withdrawing the study drug (e.g., death of a subject); the cost to the subject or health service; and the question of undue inducement if an intervention is provided free of charge.

In general, if there is good reason to believe that a product developed or knowledge generated by research is unlikely to be reasonably available to, or applied to the benefit

of, the population of a proposed host country or community after the conclusion of the research, it is unethical to conduct the research in that country or community. This should not be construed as precluding studies designed to evaluate novel therapeutic concepts. As a rare exception, for example, research may be designed to obtain preliminary evidence that a drug or a class of drugs has a beneficial effect in the treatment of a disease that occurs only in regions with extremely limited resources, and it could not be carried out reasonably well in more developed communities. Such research may be justified ethically even if there is no plan in place to make a product available to the population of the host country or community at the conclusion of the preliminary phase of its development. If the concept is found to be valid, subsequent phases of the research could result in a product that could be made reasonably available at its conclusion.

(See also Guidelines 3: *Ethical review of externally sponsored research;* 12, *Equitable distribution of burdens and benefits;* 20: *Strengthening capacity for ethical and scientific review and biomedical research;* and 21: *Ethical obligation of external sponsors to provide health-care services.)*

°

Guideline 11: Choice of control in clinical trials

As a general rule, research subjects in the control group of a trial of a diagnostic, therapeutic, or preventive intervention should receive an established effective intervention. In some circumstances it may be ethically acceptable to use an alternative comparator, such as placebo or "no treatment".

Placebo may be used:

- **when there is no established effective intervention;**
- **when withholding an established effective intervention would expose subjects to, at most, temporary discomfort or delay in relief of symptoms;**
- **when use of an established effective intervention as comparator would not yield scientifically reliable results and use of placebo would not add any risk of serious or irreversible harm to the subjects.**

Commentary on Guideline 11

General considerations for controlled clinical trials. The design of trials of investigational diagnostic, therapeutic or preventive interventions raises interrelated scientific and ethical issues for sponsors, investigators and ethical review committees. To obtain reliable results, investigators must compare the effects of an investigational intervention on subjects assigned to the investigational arm (or arms) of a trial with the effects that a control intervention produces in subjects drawn from the same population and assigned to its control arm. Randomization is the preferred method for assigning

subjects to the various arms of the clinical trial unless another method, such as historical or literature controls, can be justified scientifically and ethically. Assignment to treatment arms by randomization, in addition to its usual scientific superiority, offers the advantage of tending to render equivalent to all subjects the foreseeable benefits and risks of participation in a trial.

A clinical trial cannot be justified ethically unless it is capable of producing scientifically reliable results. When the objective is to establish the effectiveness and safety of an investigational intervention, the use of a placebo control is often much more likely than that of an active control to produce a scientifically reliable result. In many cases the ability of a trial to distinguish effective from ineffective interventions (its assay sensitivity) cannot be assured unless the control is a placebo. If, however, an effect of using a placebo would be to deprive subjects in the control arm of an established effective intervention, and thereby to expose them to serious harm, particularly if it is irreversible, it would obviously be unethical to use a placebo.

Placebo control in the absence of a current effective alternative. The use of placebo in the control arm of a clinical trial is ethically acceptable when, as stated in the Declaration of Helsinki (Paragraph 29), "no proven prophylactic, diagnostic or therapeutic method exists." Usually, in this case, a placebo is scientifically preferable to no intervention. In certain circumstances, however, an alternative design may be both scientifically and ethically acceptable, and preferable; an example would be a clinical trial of a surgical intervention, because, for many surgical interventions, either it is not possible or it is ethically unacceptable to devise a suitable placebo; for another example, in certain vaccine trials an investigator might choose to provide for those in the 'control' arm a vaccine that is unrelated to the investigational vaccine.

Placebo-controlled trials that entail only minor risks. A placebo-controlled design may be ethically acceptable, and preferable on scientific grounds, when the condition for which patients/subjects are randomly assigned to placebo or active treatment is only a small deviation in physiological measurements, such as slightly raised blood pressure or a modest increase in serum cholesterol; and if delaying or omitting available treatment may cause only temporary discomfort (e.g., common headache) and no serious adverse consequences. The ethical review committee must be fully satisfied that the risks of withholding an established effective intervention are truly minor and short-lived.

Placebo control when active control would not yield reliable results. A related but distinct rationale for using a placebo control rather than an established effective intervention is that the documented experience with the established effective intervention is not sufficient to provide a scientifically reliable comparison with the intervention being investigated; it is then difficult, or even impossible, without using a placebo, to design a scientifically reliable study. This is not always, however, an ethically acceptable basis for depriving control subjects of an established effective intervention in clinical trials; only when doing so would not add any risk of serious harm, particularly irreversible harm, to the subjects would it be ethically acceptable to do so. In some cases, the condition at which the intervention is aimed (for example, cancer or HIV/AIDS) will be too serious to deprive control subjects of an established effective intervention.

This latter rationale (*when active control would not yield reliable results)* differs from the former *(trials that entail only minor risks)* in emphasis. In trials that entail only minor risks the investigative interventions are aimed at relatively trivial conditions, such as the common cold or hair loss; forgoing an established effective intervention for the duration of a trial deprives control subjects of only minor benefits. It is for this reason that it is not unethical to use a placebo-control design. Even if it were possible to design a so-called "non-inferiority", or "equivalency", trial using an active control, it would still not be unethical in these circumstances to use a placebo-control design. In any event, the researcher must satisfy the ethical review committee that the safety and human rights of the subjects will be fully protected, that prospective subjects will be fully informed about alternative treatments, and that the purpose and design of the study are scientifically sound. The ethical acceptability of such placebo-controlled studies increases as the period of placebo use is decreased, and when the study design permits change to active treatment ("escape treatment") if intolerable symptoms occur.

Exceptional use of a comparator other than an established effective intervention. An exception to the general rule is applicable in some studies designed to develop a therapeutic, preventive or diagnostic intervention for use in a country or community in which an established effective intervention is not available and unlikely in the foreseeable future to become available, usually for economic or logistic reasons. The purpose of such a study is to make available to the population of the country or community an effective alternative to an established effective intervention that is locally unavailable. Accordingly, the proposed investigational intervention must be responsive to the health needs of the population from which the research subjects are recruited and there must be assurance that, if it proves to be safe and effective, it will be made reasonably available to that population. Also, the scientific and ethical review committees must be satisfied that the established effective intervention cannot be used as comparator because its use would not yield scientifically reliable results that would be relevant to the health needs of the study population. In these circumstances an ethical review committee can approve a clinical trial in which the comparator is other than an established effective intervention, such as placebo or no treatment or a local remedy.

However, some people strongly object to the exceptional use of a comparator other than an established effective intervention because it could result in exploitation of poor and disadvantaged populations. The objection rests on three arguments:

- Placebo control could expose research subjects to risk of serious or irreversible harm when the use of an established effective intervention as comparator could avoid the risk.

- Not all scientific experts agree about conditions under which an established effective intervention used as a comparator would not yield scientifically reliable results.

- An economic reason for the unavailability of an established effective intervention cannot justify a placebo-controlled study in a country of limited resources when it

would be unethical to conduct a study with the same design in a population with general access to the effective intervention outside the study.

Placebo control when an established effective intervention is not available in the host country. The question addressed here is: when should an exception be allowed to the general rule that subjects in the control arm of a clinical trial should receive an established effective intervention?

The usual reason for proposing the exception is that, for economic or logistic reasons, an established effective intervention is not in general use or available in the country in which the study will be conducted, whereas the investigational intervention could be made available, given the finances and infrastructure of the country.

Another reason that may be advanced for proposing a placebo-controlled trial is that using an established effective intervention as the control would not produce scientifically reliable data relevant to the country in which the trial is to be conducted. Existing data about the effectiveness and safety of the established effective intervention may have been accumulated under circumstances unlike those of the population in which it is proposed to conduct the trial**;** this, it may be argued, could make their use in the trial unreliable. One reason could be that the disease or condition manifests itself differently in different populations, or other uncontrolled factors could invalidate the use of existing data for comparative purposes.

The use of placebo control in these circumstances is ethically controversial, for the following reasons:

- Sponsors of research might use poor countries or communities as testing grounds for research that would be difficult or impossible in countries where there is general access to an established effective intervention, and the investigational intervention, if proven safe and effective, is likely to be marketed in countries in which an established effective intervention is already available and it is not likely to be marketed in the host country.

- The research subjects, both active-arm and control-arm, are patients who may have a serious, possibly life-threatening, illness. They do not normally have access to an established effective intervention currently available to similar patients in many other countries. According to the requirements of a scientifically reliable trial, investigators, who may be their attending physicians, would be expected to enrol some of those patients/subjects in the placebo-control arm. This would appear to be a violation of the physician's fiduciary duty of undivided loyalty to the patient, particularly in cases in which known effective therapy could be made available to the patients.

An argument for exceptional use of placebo control may be that a health authority in a country where an established effective intervention is not generally available or affordable, and unlikely to become available or affordable in the foreseeable future, seeks to develop an affordable intervention specifically for a health problem affecting its population. There may then be less reason for concern that a placebo design is

exploitative, and therefore unethical, as the health authority has responsibility for the population`s health, and there are valid health grounds for testing an apparently beneficial intervention. In such circumstances an ethical review committee may determine that the proposed trial is ethically acceptable, provided that the rights and safety of subjects are safeguarded.

Ethical review committees will need to engage in careful analysis of the circumstances to determine whether the use of placebo rather than an established effective intervention is ethically acceptable. They will need to be satisfied that an established effective intervention is truly unlikely to become available and implementable in that country. This may be difficult to determine, however, as it is clear that, with sufficient persistence and ingenuity, ways may be found of accessing previously unattainable medicinal products, and thus avoiding the ethical issue raised by the use of placebo control.

When the rationale of proposing a placebo-controlled trial is that the use of an established effective intervention as the control would not yield scientifically reliable data relevant to the proposed host country, the ethical review committee in that country has the option of seeking expert opinion as to whether use of an established effective intervention in the control arm would invalidate the results of the research.

An "equivalency trial" as an alternative to a placebo-controlled trial. An alternative to a placebo-control design in these circumstances would be an "equivalency trial", which would compare an investigational intervention with an established effective intervention and produce scientifically reliable data. An equivalency trial in a country in which no established effective intervention is available is not designed to determine whether the investigational intervention is superior to an established effective intervention currently used somewhere in the world; its purpose is, rather, to determine whether the investigational intervention is, in effectiveness and safety, equivalent to, or almost equivalent to, the established effective intervention. It would be hazardous to conclude, however, that an intervention demonstrated to be equivalent, or almost equivalent, to an established effective intervention is better than nothing or superior to whatever intervention is available in the country; there may be substantial differences between the results of superficially identical clinical trials carried out in different countries. If there are such differences, it would be scientifically acceptable and ethically preferable to conduct such ‘equivalency’ trials in countries in which an established effective intervention is already available.

If there are substantial grounds for the ethical review committee to conclude that an established effective intervention will not become available and implementable, the committee should obtain assurances from the parties concerned that plans have been agreed for making the investigational intervention reasonably available in the host country or community once its effectiveness and safety have been established. Moreover, when the study has external sponsorship, approval should usually be dependent on the sponsors and the health authorities of the host country having engaged in a process of negotiation and planning, including justifying the study in regard to local health-care needs.

Means of minimizing harm to placebo-control subjects. Even when placebo controls are justified on one of the bases set forth in the guideline, there are means of minimizing the possibly harmful effect of being in the control arm.

First, a placebo-control group need not be untreated. An add-on design may be employed when the investigational therapy and a standard treatment have different mechanisms of action. The treatment to be tested and placebo are each added to a standard treatment. Such studies have a particular place when a standard treatment is known to decrease mortality or irreversible morbidity but a trial with standard treatment as the active control cannot be carried out or would be difficult to interpret [*International Conference on Harmonisation* (*ICH) Guideline: Choice of Control Group and Related Issues in Clinical Trials, 2000*]. In testing for improved treatment of life-threatening diseases such as cancer, HIV/AIDS, or heart failure, add-on designs are a particularly useful means of finding improvements in interventions that are not fully effective or may cause intolerable side-effects. They have a place also in respect of treatment for epilepsy, rheumatism and osteoporosis, for example, because withholding of established effective therapy could result in progressive disability, unacceptable discomfort or both.

Second, as indicated in Guideline 8 Commentary, when the intervention to be tested in a randomized controlled trial is designed to prevent or postpone a lethal or disabling outcome, the investigator minimizes harmful effects of placebo-control studies by providing in the research protocol for the monitoring of research data by an independent Data and Safety Monitoring Board (DSMB). One function of such a board is to protect the research subjects from previously unknown adverse reactions; another is to avoid unnecessarily prolonged exposure to an inferior therapy. The board fulfils the latter function by means of interim analyses of the data pertaining to efficacy to ensure that the trial does not continue beyond the point at which an investigational therapy is demonstrated to be effective. Normally, at the outset of a randomized controlled trial, criteria are established for its premature termination (stopping rules or guidelines).

In some cases the DSMB is called upon to perform "conditional power calculations", designed to determine the probability that a particular clinical trial could ever show that the investigational therapy is effective. If that probability is very small, the DSMB is expected to recommend termination of the clinical trial, because it would be unethical to continue it beyond that point.

In most cases of research involving human subjects, it is unnecessary to appoint a DSMB. To ensure that research is carefully monitored for the early detection of adverse events, the sponsor or the principal investigator appoints an individual to be responsible for advising on the need to consider changing the system of monitoring for adverse events or the process of informed consent, or even to consider terminating the study.

◦

◦

Guideline 12: Equitable distribution of burdens and benefits in the selection of groups of subjects in research

Groups or communities to be invited to be subjects of research should be selected in such a way that the burdens and benefits of the research will be equitably distributed. The exclusion of groups or communities that might benefit from study participation must be justified.

Commentary on Guideline 12

General considerations: Equity requires that no group or class of persons should bear more than its fair share of the burdens of participation in research. Similarly, no group should be deprived of its fair share of the benefits of research, short-term or long-term; such benefits include the direct benefits of participation as well as the benefits of the new knowledge that the research is designed to yield. When burdens or benefits of research are to be apportioned unequally among individuals or groups of persons, the criteria for unequal distribution should be morally justifiable and not arbitrary. In other words, unequal allocation must not be inequitable. Subjects should be drawn from the qualifying population in the general geographic area of the trial without regard to race, ethnicity, economic status or gender unless there is a sound scientific reason to do otherwise.

In the past, groups of persons were excluded from participation in research for what were then considered good reasons. As a consequence of such exclusions, information about the diagnosis, prevention and treatment of diseases in such groups of persons is limited. This has resulted in a serious class injustice. If information about the management of diseases is considered a benefit that is distributed within a society, it is unjust to deprive groups of persons of that benefit. Such documents as the Declaration of Helsinki and the UNAIDS Guidance Document *Ethical Considerations in HIV Preventive Vaccine Research,* and the policies of many national governments and professional societies, recognize the need to redress these injustices by encouraging the participation of previously excluded groups in basic and applied biomedical research.

Members of vulnerable groups also have the same entitlement to access to the benefits of investigational interventions that show promise of therapeutic benefit as persons not considered vulnerable, particularly when no superior or equivalent approaches to therapy are available.

There has been a perception, sometimes correct and sometimes incorrect, that certain groups of persons have been overused as research subjects. In some cases such overuse has been based on the administrative availability of the populations. Research hospitals are often located in places where members of the lowest socioeconomic classes reside, and this has resulted in an apparent overuse of such persons. Other groups that may have been overused because they were conveniently available to researchers include students in investigators' classes, residents of long-term care facilities and subordinate members of hierarchical institutions. Impoverished groups have been overused because of their willingness to serve as subjects in exchange for relatively small stipends. Prisoners have been considered ideal subjects for Phase I drug studies because of their highly regimented lives and, in many cases, their conditions of economic deprivation.

Overuse of certain groups, such as the poor or the administratively available, is unjust for several reasons. It is unjust to selectively recruit impoverished people to serve as research

subjects simply because they can be more easily induced to participate in exchange for small payments. In most cases, these people would be called upon to bear the burdens of research so that others who are better off could enjoy the benefits. However, although the burdens of research should not fall disproportionately on socio-economically disadvantaged groups, neither should such groups be categorically excluded from research protocols. It would not be unjust to selectively recruit poor people to serve as subjects in research designed to address problems that are prevalent in their group – malnutrition, for example. Similar considerations apply to institutionalized groups or those whose availability to the investigators is for other reasons administratively convenient.

Not only may certain groups within a society be inappropriately overused as research subjects, but also entire communities or societies may be overused. This has been particularly likely to occur in countries or communities with insufficiently well-developed systems for the protection of the rights and welfare of human research subjects. Such overuse is especially questionable when the populations or communities concerned bear the burdens of participation in research but are extremely unlikely ever to enjoy the benefits of new knowledge and products developed as a result of the research. (See Guideline 10: *Research in populations and communities with limited resources.)*

Guideline 13: Research involving vulnerable persons

Special justification is required for inviting vulnerable individuals to serve as research subjects and, if they are selected, the means of protecting their rights and welfare must be strictly applied.

Commentary on Guideline 13

Vulnerable persons are those who are relatively (or absolutely) incapable of protecting their own interests. More formally, they may have insufficient power, intelligence, education, resources, strength, or other needed attributes to protect their own interests.

General considerations. The central problem presented by plans to involve vulnerable persons as research subjects is that such plans may entail an inequitable distribution of the burdens and benefits of research participation. Classes of individuals conventionally considered vulnerable are those with limited capacity or freedom to consent or to decline to consent. They are the subject of specific guidelines in this document (Guidelines 14,15) and include children, and persons who because of mental or behavioural disorders are incapable of giving informed consent. Ethical justification of their involvement usually requires that investigators satisfy ethical review committees that:

- the research could not be carried out equally well with less vulnerable subjects;

- the research is intended to obtain knowledge that will lead to improved diagnosis, prevention or treatment of diseases or other health problems characteristic of, or

unique to, the vulnerable class– either the actual subjects or other similarly situated members of the vulnerable class;

- research subjects and other members of the vulnerable class from which subjects are recruited will ordinarily be assured reasonable access to any diagnostic, preventive or therapeutic products that will become available as a consequence of the research;

- the risks attached to interventions or procedures that do not hold out the prospect of direct health-related benefit will not exceed those associated with routine medical or psychological examination of such persons unless an ethical review committee authorizes a slight increase over this level of risk (Guideline 9); and,

- when the prospective subjects are either incompetent or otherwise substantially unable to give informed consent, their agreement will be supplemented by the permission of their legal guardians or other appropriate representatives.

Other vulnerable groups. The quality of the consent of prospective subjects who are junior or subordinate members of a hierarchical group requires careful consideration, as their agreement to volunteer may be unduly influenced, whether justified or not, by the expectation of preferential treatment if they agree or by fear of disapproval or retaliation if they refuse. Examples of such groups are medical and nursing students, subordinate hospital and laboratory personnel, employees of pharmaceutical companies, and members of the armed forces or police. Because they work in close proximity to investigators, they tend to be called upon more often than others to serve as research subjects, and this could result in inequitable distribution of the burdens and benefits of research.

Elderly persons are commonly regarded as vulnerable. With advancing age**,** people are increasingly likely to acquire attributes that define them as vulnerable. They may, for example, be institutionalized or develop varying degrees of dementia**.** If and when they acquire such vulnerability-defining attributes, and not before, it is appropriate to consider them vulnerable and to treat them accordingly.

Other groups or classes may also be considered vulnerable. They include residents of nursing homes, people receiving welfare benefits or social assistance and other poor people and the unemployed, patients in emergency rooms, some ethnic and racial minority groups, homeless persons, nomads, refugees or displaced persons, prisoners, patients with incurable disease, individuals who are politically powerless, and members of communities unfamiliar with modern medical concepts. To the extent that these and other classes of people have attributes resembling those of classes identified as vulnerable, the need for special protection of their rights and welfare should be reviewed and applied, where relevant.

Persons who have serious, potentially disabling or life-threatening diseases are highly vulnerable**.** Physicians sometimes treat such patients with drugs or other therapies not yet licensed for general availability because studies designed to establish their safety and efficacy have not been completed. This is compatible with the Declaration of Helsinki,

which states in Paragraph 32*: " In the treatment of a patient, where proven therapeutic methods do not exist or have been ineffective, the physician, with informed consent from the patient, must be free to use unproven or new therapeutic measures, if in the physician s judgement it offers hope of saving life, re-establishing health or alleviating suffering"*. Such treatment, commonly called 'compassionate use', is not properly regarded as research, but it can contribute to ongoing research into the safety and efficacy of the interventions used.

Although, on the whole, investigators must study less vulnerable groups before involving more vulnerable groups, some exceptions are justified. In general, children are not suitable for Phase I drug trials or for Phase I or II vaccine trials, but such trials may be permissible after studies in adults have shown some therapeutic or preventive effect. For example, a Phase II vaccine trial seeking evidence of immunogenicity in infants may be justified when a vaccine has shown evidence of preventing or slowing progression of an infectious disease in adults, or Phase I research with children may be appropriate because the disease to be treated does not occur in adults or is manifested differently in children (Appendix 3: *The phases of clinical trials of vaccines and drugs*).

°

Guideline 14: Research involving children

Before undertaking research involving children, the investigator must ensure that:

- **the research might not equally well be carried out with adults;**
- **the purpose of the research is to obtain knowledge relevant to the health needs of children;**
- **a parent or legal representative of each child has given permission;**
- **the agreement (assent) of each child has been obtained to the extent of the child's capabilities; and,**
- **a child's refusal to participate or continue in the research will be respected.**

Commentary on Guideline 14

Justification of the involvement of children in biomedical research. The participation of children is indispensable for research into diseases of childhood and conditions to which children are particularly susceptible (cf. vaccine trials), as well as for clinical trials of drugs that are designed for children as well as adults. In the past, many new products were not tested for children though they were directed towards diseases also occurring in childhood; thus children either did not benefit from these new drugs or were exposed to them though little was known about their specific effects or safety in children. Now it is widely agreed that, as a general rule, the sponsor of any new therapeutic, diagnostic or

preventive product that is likely to be indicated for use in children is obliged to evaluate its safety and efficacy for children before it is released for general distribution.

Assent of the child. The willing cooperation of the child should be sought, after the child has been informed to the extent that the child's maturity and intelligence permit. The age at which a child becomes legally competent to give consent differs substantially from one jurisdiction to another; in some countries the "age of consent" established in their different provinces, states or other political subdivisions varies considerably. Often children who have not yet reached the legally established age of consent can understand the implications of informed consent and go through the necessary procedures; they can therefore knowingly agree to serve as research subjects. Such knowing agreement, sometimes referred to as assent, is insufficient to permit participation in research unless it is supplemented by the permission of a parent, a legal guardian or other duly authorized representative.

Some children who are too immature to be able to give knowing agreement, or assent, may be able to register a 'deliberate objection', an expression of disapproval or refusal of a proposed procedure. The deliberate objection of an older child, for example, is to be distinguished from the behaviour of an infant, who is likely to cry or withdraw in response to almost any stimulus. Older children, who are more capable of giving assent, should be selected before younger children or infants, unless there are valid scientific reasons related to age for involving younger children first.

A deliberate objection by a child to taking part in research should always be respected even if the parents have given permission, unless the child needs treatment that is not available outside the context of research, the investigational intervention shows promise of therapeutic benefit, and there is no acceptable alternative therapy. In such a case, particularly if the child is very young or immature, a parent or guardian may override the child`s objections. If the child is older and more nearly capable of independent informed consent, the investigator should seek the specific approval or clearance of the scientific and ethical review committees for initiating or continuing with the investigational treatment. If child subjects become capable of independent informed consent during the research, their informed consent to continued participation should be sought and their decision respected.

A child with a likely fatal illness may object or refuse assent to continuation of a burdensome or distressing intervention. In such circumstances parents may press an investigator to persist with an investigational intervention against the child`s wishes. The investigator may agree to do so if the intervention shows promise of preserving or prolonging life and there is no acceptable alternative treatment. In such cases, the investigator should seek the specific approval or clearance of the ethical review committee before agreeing to override the wishes of the child.

Permission of a parent or guardian. The investigator must obtain the permission of a parent or guardian in accordance with local laws or established procedures. It may be assumed that children over the age of 12 or 13 years are usually capable of understanding what is necessary to give adequately informed consent, but their consent (assent) should normally be complemented by the permission of a parent or guardian, even when local

law does not require such permission. Even when the law requires parental permission, however, the assent of the child must be obtained.

In some jurisdictions, some individuals who are below the general age of consent are regarded as "emancipated" or "mature" minors and are authorized to consent without the agreement or even the awareness of their parents or guardians. They may be married or pregnant or be already parents or living independently. Some studies involve investigation of adolescents' beliefs and behaviour regarding sexuality or use of recreational drugs; other research addresses domestic violence or child abuse. For studies on these topics, ethical review committees may waive parental permission if, for example, parental knowledge of the subject matter may place the adolescents at some risk of questioning or even intimidation by their parents.

Because of the issues inherent in obtaining assent from children in institutions, such children should only exceptionally be subjects of research. In the case of institutionalized children without parents, or whose parents are not legally authorized to grant permission, the ethical review committee may require sponsors or investigators to provide it with the opinion of an independent, concerned, expert advocate for institutionalized children as to the propriety of undertaking the research with such children.

Observation of research by a parent or guardian. A parent or guardian who gives permission for a child to participate in research should be given the opportunity, to a reasonable extent, to observe the research as it proceeds, so as to be able to withdraw the child if the parent or guardian decides it is in the child's best interests to do so.

Psychological and medical support. Research involving children should be conducted in settings in which the child and the parent can obtain adequate medical and psychological support. As an additional protection for children, an investigator may, when possible, obtain the advice of a child's family physician, paediatrician or other health-care provider on matters concerning the child's participation in the research.

(See also Guideline 8: *Benefits and risks of study participation*; Guideline 9: *Special limitations on risks when subjects are not capable of giving consent*; and Guideline 13: *Research involving vulnerable persons.)*

Guideline 15: Research involving individuals who by reason of mental or behavioural disorders are not capable of giving adequately informed consent

Before undertaking research involving individuals who by reason of mental or behavioural disorders are not capable of giving adequately informed consent, the investigator must ensure that:

- **such persons will not be subjects of research that might equally well be carried out on persons whose capacity to give adequately informed consent is not impaired;**

- **the purpose of the research is to obtain knowledge relevant to the particular health needs of persons with mental or behavioural disorders;**

- **the consent of each subject has been obtained to the extent of that person's capabilities, and a prospective subject's refusal to participate in research is always respected, unless, in exceptional circumstances, there is no reasonable medical alternative and local law permits overriding the objection; and,**

- **in cases where prospective subjects lack capacity to consent, permission is obtained from a responsible family member or a legally authorized representative in accordance with applicable law.**

Commentary on Guideline 15

General considerations. Most individuals with mental or behavioural disorders are capable of giving informed consent; this Guideline is concerned only with those who are not capable or who because their condition deteriorates become temporarily incapable. They should never be subjects of research that might equally well be carried out on persons in full possession of their mental faculties, but they are clearly the only subjects suitable for a large part of research into the origins and treatment of certain severe mental or behavioural disorders.

Consent of the individual. The investigator must obtain the approval of an ethical review committee to include in research persons who by reason of mental or behavioural disorders are not capable of giving informed consent. The willing cooperation of such persons should be sought to the extent that their mental state permits, and any objection on their part to taking part in any study that has no components designed to benefit them directly should always be respected. The objection of such an individual to an investigational intervention intended to be of therapeutic benefit should be respected unless there is no reasonable medical alternative and local law permits overriding the objection. The agreement of an immediate family member or other person with a close personal relationship with the individual should be sought, but it should be recognized that these proxies may have their own interests that may call their permission into question. Some relatives may not be primarily concerned with protecting the rights and welfare of the patients. Moreover, a close family member or friend may wish to take advantage of a research study in the hope that it will succeed in "curing" the condition. Some jurisdictions do not permit third-party permission for subjects lacking capacity to consent.Legal authorization may be necessary to involve in research an individual who has been committed to an institution by a court order.

Serious illness in persons who because of mental or behavioural disorders are unable to give adequately informed consent. Persons who because of mental or behavioural disorders are unable to give adequately informed consent and who have, or are at risk of, serious illnesses such as HIV infection, cancer or hepatitis should not be deprived of the possible benefits of investigational drugs, vaccines or devices that show promise of therapeutic or preventive benefit, particularly when no superior or equivalent therapy or

prevention is available. Their entitlement to access to such therapy or prevention is justified ethically on the same grounds as is such entitlement for other vulnerable groups.

Persons who are unable to give adequately informed consent by reason of mental or behavioural disorders are, in general, not suitable for participation in formal clinical trials except those trials that are designed to be responsive to their particular health needs and can be carried out only with them.

(See also Guidelines 8: *Benefits and risks of study participation;* 9: *Special limitations on risks when subjects are not capable of giving consent;* and 13: *Research involving vulnerable persons*.)

Guideline 16: Women as research subjects

Investigators, sponsors or ethical review committees should not exclude women of reproductive age from biomedical research. The potential for becoming pregnant during a study should not, in itself, be used as a reason for precluding or limiting participation. However, a thorough discussion of risks to the pregnant woman and to her fetus is a prerequisite for the woman s ability to make a rational decision to enrol in a clinical study. In this discussion, if participation in the research might be hazardous to a fetus or a woman if she becomes pregnant, the sponsors/ investigators should guarantee the prospective subject a pregnancy test and access to effective contraceptive methods before the research commences. Where such access is not possible, for legal or religious reasons, investigators should not recruit for such possibly hazardous research women who might become pregnant.

Commentary on Guideline 16

Women in most societies have been discriminated against with regard to their involvement in research. Women who are biologically capable of becoming pregnant have been customarily excluded from formal clinical trials of drugs, vaccines and medical devices owing to concern about undetermined risks to the fetus. Consequently, relatively little is known about the safety and efficacy of most drugs, vaccines or devices for such women, and this lack of knowledge can be dangerous.

A general policy of excluding from such clinical trials women biologically capable of becoming pregnant is unjust in that it deprives women as a class of persons of the benefits of the new knowledge derived from the trials. Further, it is an affront to their right of self-determination. Nevertheless, although women of childbearing age should be given the opportunity to participate in research, they should be helped to understand that the research could include risks to the fetus if they become pregnant during the research.

Although this general presumption favours the inclusion of women in research, it must be acknowledged that in some parts of the world women are vulnerable to neglect or harm in research because of their social conditioning to submit to authority, to ask no questions,

and to tolerate pain and suffering. When women in such situations are potential subjects in research, investigators need to exercise special care in the informed consent process to ensure that they have adequate time and a proper environment in which to take decisions on the basis of clearly given information.

Individual consent of women: In research involving women of reproductive age, whether pregnant or non-pregnant, only the informed consent of the woman herself is required for her participation. In no case should the permission of a spouse or partner replace the requirement of individual informed consent. If women wish to consult with their husbands or partners or seek voluntarily to obtain their permission before deciding to enrol in research, that is not only ethically permissible but in some contexts highly desirable. A strict requirement of authorization of spouse or partner, however, violates the substantive principle of respect for persons.

A thorough discussion of risks to the pregnant woman and to her fetus is a prerequisite for the woman's ability to make a rational decision to enrol in a clinical study. For women who are not pregnant at the outset of a study but who might become pregnant while they are still subjects, the consent discussion should include information about the alternative of voluntarily withdrawing from the study and, where legally permissible, terminating the pregnancy. Also, if the pregnancy is not terminated, they should be guaranteed a medical follow-up.

°

Guideline 17: Pregnant women as research participants.

Pregnant women should be presumed to be eligible for participation in biomedical research. Investigators and ethical review committees should ensure that prospective subjects who are pregnant are adequately informed about the risks and benefits to themselves, their pregnancies, the fetus and their subsequent offspring, and to their fertility.

Research in this population should be performed only if it is relevant

to the particular health needs of a pregnant woman or her fetus, or to the health needs of pregnant women in general, and, when appropriate, if it is supported by reliable evidence from animal experiments, particularly as to risks of teratogenicity and mutagenicity .

Commentary on Guideline 17

The justification of research involving pregnant women is complicated by the fact that it may present risks and potential benefits to two beings – the woman and the fetus – as well as to the person the fetus is destined to become. Though the decision about acceptability of risk should be made by the mother as part of the informed consent process, it is desirable in research directed at the health of the fetus to obtain the father´s opinion also, when possible. Even when evidence concerning risks is unknown or

ambiguous, the decision about acceptability of risk to the fetus should be made by the woman as part of the informed consent process.

Especially in communities or societies in which cultural beliefs accord more importance to the fetus than to the woman's life or health, women may feel constrained to participate, or not to participate, in research. Special safeguards should be established to prevent undue inducement to pregnant women to participate in research in which interventions hold out the prospect of direct benefit to the fetus. Where fetal abnormality is not recognized as an indication for abortion, pregnant women should not be recruited for research in which there is a realistic basis for concern that fetal abnormality may occur as a consequence of participation as a subject in research.

Investigators should include in protocols on research on pregnant women a plan for monitoring the outcome of the pregnancy with regard to both the health of the woman and the short-term and long-term health of the child.

Guideline 18: Safeguarding confidentiality

The investigator must establish secure safeguards of the confidentiality of subjects research data. Subjects should be told the limits, legal or other, to the investigators' ability to safeguard confidentiality and the possible consequences of breaches of confidentiality.

Commentary on Guideline 18

Confidentiality between investigator and subject. Research relating to individuals and groups may involve the collection and storage of information that, if disclosed to third parties, could cause harm or distress. Investigators should arrange to protect the confidentiality of such information by, for example, omitting information that might lead to the identification of individual subjects, limiting access to the information, anonymizing data, or other means. During the process of obtaining informed consent the investigator should inform the prospective subjects about the precautions that will be taken to protect confidentiality.

Prospective subjects should be informed of limits to the ability of investigators to ensure strict confidentiality and of the foreseeable adverse social consequences of breaches of confidentiality. Some jurisdictions require the reporting to appropriate agencies of, for instance, certain communicable diseases or evidence of child abuse or neglect. Drug regulatory authorities have the right to inspect clinical-trial records, and a sponsor`s clinical-compliance audit staff may require and obtain access to confidential data. These and similar limits to the ability to maintain confidentiality should be anticipated and disclosed to prospective subjects**.**

Participation in HIV/AIDS drug and vaccine trials may impose upon the research subjects significant associated risks of social discrimination or harm; such risks merit

consideration equal to that given to adverse medical consequences of the drugs and vaccines. Efforts must be made to reduce their likelihood and severity. For example, subjects in vaccine trials must be enabled to demonstrate that their HIV seropositivity is due to their having been vaccinated rather than to natural infection. This may be accomplished by providing them with documents attesting to their participation in vaccine trials, or by maintaining a confidential register of trial subjects, from which information can be made available to outside agencies at a subject's request.

Confidentiality between physician and patient. Patients have the right to expect that their physicians and other health-care professionals will hold all information about them in strict confidence and disclose it only to those who need, or have a legal right to, the information, such as other attending physicians, nurses, or other health-care workers who perform tasks related to the diagnosis and treatment of patients. A treating physician should not disclose any identifying information about patients to an investigator unless each patient has given consent to such disclosure and unless an ethical review committee has approved such disclosure.

Physicians and other health care professionals record the details of their observations and interventions in medical and other records. Epidemiological studies often make use of such records. For such studies it is usually impracticable to obtain the informed consent of each identifiable patient; an ethical review committee may waive the requirement for informed consent when this is consistent with the requirements of applicable law and provided that there are secure safeguards of confidentiality. (See also Guideline 4 Commentary: *Waiver of the consent requirement.)* In institutions in which records may be used for research purposes without the informed consent of patients, it is advisable to notify patients generally of such practices; notification is usually by means of a statement in patient-information brochures. For research limited to patients' medical records, access must be approved or cleared by an ethical review committee and must be supervised by a person who is fully aware of the confidentiality requirements.

Issues of confidentiality in genetic research. An investigator who proposes to perform genetic tests of known clinical or predictive value on biological samples that can be linked to an identifiable individual must obtain the informed consent of the individual or, when indicated, the permission of a legally authorized representative. Conversely, before performing a genetic test that is of known predictive value or gives reliable information about a known heritable condition, and individual consent or permission has not been obtained, investigators must see that biological samples are fully anonymized and unlinked; this ensures that no information about specific individuals can be derived from such research or passed back to them.

When biological samples are not fully anonymized and when it is anticipated that there may be valid clinical or research reasons for linking the results of genetic tests to research subjects, the investigator in seeking informed consent should assure prospective subjects that their identity will be protected by secure coding of their samples (encryption) and by restricted access to the database, and explain to them this process.

When it is clear that for medical or possibly research reasons the results of genetic tests will be reported to the subject or to the subject`s physician, the subject should be

informed that such disclosure will occur and that the samples to be tested will be clearly labelled.

Investigators should not disclose results of diagnostic genetic tests to relatives of subjects without the subjects` consent. In places where immediate family relatives would usually expect to be informed of such results, the research protocol, as approved or cleared by the ethical review committee, should indicate the precautions in place to prevent such disclosure of results without the subjects`consent; such plans should be clearly explained during the process of obtaining informed consent.

Guideline 19: Right of injured subjects to treatment and compensation

Investigators should ensure that research subjects who suffer injury as a result of their participation are entitled to free medical treatment for such injury and to such financial or other assistance as would compensate them equitably for any resultant impairment, disability or handicap. In the case of death as a result of their participation, their dependants are entitled to compensation. Subjects must not be asked to waive the right to compensation.

Commentary on Guideline 19

Guideline 19 is concerned with two distinct but closely related entitlements. The first is the uncontroversial entitlement to free medical treatment and compensation for accidental injury inflicted by procedures or interventions performed exclusively to accomplish the purposes of research (non-therapeutic procedures). The second is the entitlement of dependants to material compensation for death or disability occurring as a direct result of study participation. Implementing a compensation system for research-related injuries or death is likely to be complex, however.

Equitable compensation and free medical treatment. Compensation is owed to research subjects who are disabled as a consequence of injury from procedures performed solely to accomplish the purposes of research. Compensation and free medical treatment are generally not owed to research subjects who suffer expected or foreseen adverse reactions to investigational therapeutic, diagnostic or preventive interventions when such reactions are not different in kind from those known to be associated with established interventions in standard medical practice. In the early stages of drug testing (Phase I and early Phase II), it is generally unreasonable to assume that an investigational drug holds out the prospect of direct benefit for the individual subject; accordingly, compensation is usually owed to individuals who become disabled as a result of serving as subjects in such studies.

The ethical review committee should determine in advance: i) the injuries for which subjects will receive free treatment and, in case of impairment, disability or handicap resulting from such injuries, be compensated; and ii) the injuries for which they will not be compensated. Prospective subjects should be informed of the committee's decisions, as

part of the process of informed consent. As an ethical review committee cannot make such advance determination in respect of unexpected or unforeseen adverse reactions, such reactions must be presumed compensable and should be reported to the committee for prompt review as they occur.

Subjects must not be asked to waive their rights to compensation or required to show negligence or lack of a reasonable degree of skill on the part of the investigator in order to claim free medical treatment or compensation. The informed consent process or form should contain no words that would absolve an investigator from responsibility in the case of accidental injury, or that would imply that subjects would waive their right to seek compensation for impairment, disability or handicap. Prospective subjects should be informed that they will not need to take legal action to secure the free medical treatment or compensation for injury to which they may be entitled. They should also be told what medical service or organization or individual will provide the medical treatment and what organization will be responsible for providing compensation.

Obligation of the sponsor with regard to compensation. Before the research begins, the sponsor, whether a pharmaceutical company or other organization or institution, or a government (where government insurance is not precluded by law), should agree to provide compensation for any physical injury for which subjects are entitled to compensation, or come to an agreement with the investigator concerning the circumstances in which the investigator must rely on his or her own insurance coverage (for example, for negligence or failure of the investigator to follow the protocol, or where government insurance coverage is limited to negligence). In certain circumstances it may be advisable to follow both courses. Sponsors should seek adequate insurance against risks to cover compensation, independent of proof of fault.

Guideline 20: Strengthening capacity for ethical and scientific review and biomedical research

Many countries lack the capacity to assess or ensure the scientific quality or ethical acceptability of biomedical research proposed or carried out in their jurisdictions. In externally sponsored collaborative research, sponsors and investigators have an ethical obligation to ensure that biomedical research projects for which they are responsible in such countries contribute effectively to national or local capacity to design and conduct biomedical research, and to provide scientific and ethical review and monitoring of such research.

Capacity-building may include, but is not limited to, the following activities:

- **establishing and strengthening independent and competent ethical review processes/ committees**

- **strengthening research capacity**

- **developing technologies appropriate to health-care and biomedical research**
- **training of research and health-care staff**
- **educating the community from which research subjects will be drawn**

Commentary on Guideline 20

External sponsors and investigators have an ethical obligation to contribute to a host country's sustainable capacity for independent scientific and ethical review and biomedical research. Before undertaking research in a host country with little or no such capacity, external sponsors and investigators should include in the research protocol a plan that specifies the contribution they will make. The amount of capacity building reasonably expected should be proportional to the magnitude of the research project. A brief epidemiological study involving only review of medical records, for example, would entail relatively little, if any, such development, whereas a considerable contribution is to be expected of an external sponsor of, for instance, a large-scale vaccine field-trial expected to last two or three years.

The specific capacity-building objectives should be determined and achieved through dialogue and negotiation between external sponsors and host-country authorities. External sponsors would be expected to employ and, if necessary, train local individuals to function as investigators, research assistants or data managers, for example, and to provide, as necessary, reasonable amounts of financial, educational and other assistance for capacity-building. To avoid conflict of interest and safeguard the independence of review committees, financial assistance should not be provided directly to them; rather, funds should be made available to appropriate authorities in the host-country government or to the host research institution.

(See also Guideline 10: *Research in populations and communities with limited resources*)

Guideline 21: Ethical obligation of external sponsors to provide health-care services

External sponsors are ethically obliged to ensure the availability of:

°

– health-care services that are essential to the safe conduct of the research;

– treatment for subjects who suffer injury as a consequence of research interventions; and,

– services that are a necessary part of the commitment of a sponsor to make a beneficial intervention or product developed as a result of the research reasonably available to the population or community concerned.

Commentary on Guideline 21

Obligations of external sponsors to provide health-care services will vary with the circumstances of particular studies and the needs of host countries. The sponsors' obligations in particular studies should be clarified before the research is begun. The research protocol should specify what health-care services will be made available, during and after the research, to the subjects themselves, to the community from which the subjects are drawn, or to the host country, and for how long. The details of these arrangements should be agreed by the sponsor, officials of the host country, other interested parties, and, when appropriate, the community from which subjects are to be drawn. The agreed arrangements should be specified in the consent process and document.

Although sponsors are, in general, not obliged to provide health-care services beyond that which is necessary for the conduct of the research, it is morally praiseworthy to do so. Such services typically include treatment for diseases contracted in the course of the study. It might, for example, be agreed to treat cases of an infectious disease contracted during a trial of a vaccine designed to provide immunity to that disease, or to provide treatment of incidental conditions unrelated to the study.

The obligation to ensure that subjects who suffer injury as a consequence of research interventions obtain medical treatment free of charge, and that compensation be provided for death or disability occurring as a consequence of such injury, is the subject of Guideline 19, on the scope and limits of such obligations.

When prospective or actual subjects are found to have diseases unrelated to the research, or cannot be enrolled in a study because they do not meet the health criteria, investigators should, as appropriate, advise them to obtain, or refer them for, medical care. In general, also, in the course of a study, sponsors should disclose to the proper health authorities information of public health concern arising from the research.

The obligation of the sponsor to make reasonably available for the benefit of the population or community concerned any intervention or product developed, or knowledge generated, as a result of the research is considered in Guideline 10: *Research in populations and communities with limited resources.*

◦

◦

Appendix 1

Items to be included in a protocol (or associated documents) for biomedical research involving human subjects.

(Include the items relevant to the study/project in question)

1. Title of the study;

2. A summary of the proposed research in lay/non-technical language.

3. A clear statement of the justification for the study, its significance in development and in meeting the needs of the country /population in which the research is carried out;

4. The investigators` views of the ethical issues and considerations raised by the study and, if appropriate, how it is proposed to deal with them;

5. Summary of all previous studies on the topic, including unpublished studies known to the investigators and sponsors, and information on previously published research on the topic, including the nature, extent and relevance of animal studies and other preclinical and clinical studies;

6. A statement that the principles set out in these Guidelines will be implemented;

7. An account of previous submissions of the protocol for ethical review and their outcome;

8. A brief description of the site(s) where the research is to be conducted, including information about the adequacy of facilities for the safe and appropriate conduct of the research, and *relevant* demographic and epidemiological information about the country or region concerned;

9. Name and address of the sponsor;

10. Names, address*es*, institutional affiliations*,* qualifications and experience of the principal investigator and other investigators;

11. The objectives of the trial or study, its hypotheses or research questions, its assumptions, and its variables;

12. A detailed description of the design of the trial or study. In the case of controlled clinical trials the description should include, but not be limited to, whether assignment to treatment groups will be randomized (including the method of randomization), and whether the study will be blinded (single blind, double blind), or open;

13. The number of research subjects needed to achieve the study objective, and how this was statistically determined;

14. The criteria for inclusion or exclusion of potential subjects, and justification for the exclusion of any groups on the basis of age, sex, social or economic factors, or for other reasons;

15. The justification for involving as research subjects any persons with limited capacity to consent or members of vulnerable social groups, and a description of special measures to minimize risks and discomfort to such subjects;

16. The process of recruitment, e.g., advertisements, and the steps to be taken to protect privacy and confidentiality during recruitment;

17. Description and explanation of all interventions (the method of treatment administration, including route of administration, dose, dose interval and treatment period for investigational and comparator products used);

18. Plans and justification for withdrawing or withholding standard therapies in the course of the research, including any resulting risks to subjects;

19. Any other treatment that may be given or permitted, or contraindicated, during the study;

20. Clinical and laboratory tests and other tests that are to be carried out;

21. Samples of the standardized case-report forms to be used, the methods of recording therapeutic response (description and evaluation of methods and frequency of measurement), the follow-up procedures, and, if applicable, the measures proposed to determine the extent of compliance of subjects with the treatment;

22. Rules or criteria according to which subjects may be removed from the study or clinical trial, or (in a multi-centre study) a centre may be discontinued, or the study may be terminated;

23. Methods of recording and reporting adverse events or reactions, and provisions for dealing with complications;

24. The known or foreseen risks of adverse reactions, including the risks attached to each proposed intervention and to any drug, vaccine or procedure to be tested;

25. For research carrying more than minimal risk of physical injury, details of plans, including insurance coverage, to provide treatment for such injury, including the funding of treatment, and to provide compensation for research-related disability or death;

26. Provision for continuing access of subjects to the investigational treatment after the study, indicating its modalities, the individual or organization responsible for paying for it, and for how long it will continue;

27. For research on pregnant women, a plan, if appropriate, for monitoring the outcome of the pregnancy with regard to both the health of the woman and the short-term and long-term health of the child.

28. The potential benefits of the research to subjects and to others;

29. The expected benefits of the research to the population, including new knowledge that the study might generate;

30. The means proposed to obtain individual informed consent and the procedure planned to communicate information to prospective subjects, including the name and position of the person responsible for obtaining consent;

31. When a prospective subject is not capable of informed consent, satisfactory assurance that permission will be obtained from a duly authorized person, or, in the case of a child who is sufficiently mature to understand the implications of informed consent but has not reached the legal age of consent, that knowing agreement, or assent, will be obtained, as well as the permission of a parent, or a legal guardian or other duly authorized representative;

32. An account of any economic or other inducements or incentives to prospective subjects to participate, such as offers of cash payments, gifts, or free services or facilities, and of any financial obligations assumed by the subjects, such as payment for medical services;

33. Plans and procedures, and the persons responsible, for communicating to subjects information arising from the study (on harm or benefit, for example), or from other research on the same topic, that could affect subjects' willingness to continue in the study;

34. Plans to inform subjects about the results of the study;

35. The provisions for protecting the confidentiality of personal data, and respecting the privacy of subjects, including the precautions that are in place to prevent disclosure of the results of a subject's genetic tests to immediate family relatives without the consent of the subject;

36. Information about how the code, if any, for the subjects' identity is established, where it will be kept and when, how and by whom it can be broken in the event of an emergency;

37. Any foreseen further uses of personal data or biological materials;

38. A description of the plans for statistical analysis of the study, including plans for interim analyses, if any, and criteria for prematurely terminating the study as a whole if necessary;

39. Plans for monitoring the continuing safety of drugs or other interventions administered for purposes of the study or trial and, if appropriate, the appointment for this purpose of an independent data-monitoring (data and safety monitoring) committee;

40. A list of the references cited in the protocol;

41. The source and amount of funding of the research: the organization that is sponsoring the research and a detailed account of the sponsor's financial commitments to the research institution, the investigators, the research subjects, and, when relevant, the community;

42. The arrangements for dealing with financial or other conflicts of interest that might affect the judgement of investigators or other research personnel: informing the institutional conflict-of-interest committee of such conflicts of interest; the communication by that committee of the pertinent details of the information to the ethical review committee; and the transmission by that committee to the research subjects of the parts of the information that it decides should be passed on to them;

43. The time schedule for completion of the study;

44. For research that is to be carried out in a developing country or community, the contribution that the sponsor will make to capacity-building for scientific and ethical review and for biomedical research in the host country, and an assurance that the capacity-building objectives are in keeping with the values and expectations of the subjects and their communities;

45. Particularly in the case of an industrial sponsor, a contract stipulating who possesses the right to publish the results of the study, and a mandatory obligation to prepare with, and submit to, the principal investigators the draft of the text reporting the results;

46. In the case of a negative outcome, an assurance that the results will be made available, as appropriate, through publication or by reporting to the drug registration authority;

47. Circumstances in which it might be considered inappropriate to publish findings, such as when the findings of an epidemiological, sociological or genetics study may present risks to the interests of a community or population or of a racially or ethnically defined group of people;

48. A statement that any proven evidence of falsification of data will be dealt with in accordance with the policy of the sponsor to take appropriate action against such unacceptable procedures.

Appendix 2

WORLD MEDICAL ASSOCIATION DECLARATION OF HELSINKI

<www.wma.net>

Appendix 3

THE PHASES OF CLINICAL TRIALS OF VACCINES AND DRUGS

Vaccine development

Phase I refers to the first introduction of a candidate vaccine into a human population for initial determination of its safety and biological effects, including immunogenicity. This phase may include studies of dose and route of administration, and usually involves fewer than 100 volunteers.

Phase II refers to the initial trials examining effectiveness in a limited number of volunteers (usually between 200 and 500); the focus of this phase is immunogenicity.

Phase III trials are intended for a more complete assessment of safety and effectiveness in the prevention of disease, involving a larger number of volunteers in a multicentre adequately controlled study.

Drug development

Phase I refers to the first introduction of a drug into humans. Normal volunteer subjects are usually studied to determine levels of drugs at which toxicity is observed. Such studies are followed by dose-ranging studies in patients for safety and, in some cases, early evidence of effectiveness.

Phase II investigation consists of controlled clinical trials designed to demonstrate effectiveness and relative safety. Normally, these are performed on a limited number of closely monitored patients.

Phase III trials are performed after a reasonable probability of effectiveness of a drug has been established and are intended to gather additional evidence of effectiveness for specific indications and more precise definition of drug-related adverse effects. This phase includes both controlled and uncontrolled studies.

Phase II and Phase III drug trials should be conducted according to Section C (Paragraphs 28–32) of the Declaration of Helsinki, which refers to medical research combined with medical care.

Phase IV trials are conducted after the national drug registration authority has approved a drug for distribution or marketing. These trials may include research designed to explore a specific pharmacological effect, to establish the incidence of adverse reactions, or to determine the effects of long-term administration of a drug. Phase IV trials may also be designed to evaluate a drug in a population not studied adequately in the pre-marketing phases (such as children or the elderly) or to establish a new clinical indication for a drug. Such research is to be distinguished from marketing research, sales promotion studies, and routine post-marketing surveillance for adverse drug reactions in that these categories ordinarily need not be reviewed by ethical review committees (see Guideline 2).
